# Evolution of Molecular Targeted Cancer Therapy: Mechanisms of Drug Resistance and Novel Opportunities Identified by CRISPR-Cas9 Screening

**DOI:** 10.3389/fonc.2022.755053

**Published:** 2022-03-17

**Authors:** Jue Hou, Zongsheng He, Tian Liu, Dongfeng Chen, Bin Wang, Qinglian Wen, Xi Zheng

**Affiliations:** ^1^ Department of Oncology, The Affiliated Hospital of Southwest Medical University, Luzhou, China; ^2^ Department of Gastroenterology, Chongqing Key Laboratory of Digestive Malignancies, Daping Hospital, Army Medical University (Third Military Medical University), Chongqing, China; ^3^ Department of Gastroenterology, Chongqing University Cancer Hospital, Chongqing, China

**Keywords:** molecular targeted therapy, drug resistance, CRISPR-Cas9 screening, patient-derived xenograft (PDX), patient-derived organoid

## Abstract

Molecular targeted therapy has revolutionized the landscape of cancer treatment due to better therapeutic responses and less systemic toxicity. However, therapeutic resistance is a major challenge in clinical settings that hinders continuous clinical benefits for cancer patients. In this regard, unraveling the mechanisms of drug resistance may identify new druggable genetic alterations for molecularly targeted therapies, thus contributing to improved therapeutic efficacies. The recent rapid development of novel methodologies including CRISPR-Cas9 screening technology and patient-derived models provides powerful tools to dissect the underlying mechanisms of resistance to targeted cancer therapies. In this review, we updated therapeutic targets undergoing preclinical and clinical evaluation for various cancer types. More importantly, we provided comprehensive elaboration of high throughput CRISPR-Cas9 screening in deciphering potential mechanisms of unresponsiveness to molecularly targeted therapies, which will shed light on the discovery of novel opportunities for designing next-generation anti-cancer drugs.

## 1 Introduction

Transformation of normal human cells into malignant states is driven by multistep alterations of genes. Of these alterations, the majority of them are largely neutral (passenger mutations) in comparison to a few driver mutations that endow cells with tumorigenic properties which can be fractionized as six parts: sustaining proliferative signaling, evading growth suppressors, resisting cell death, inducing angiogenesis, activating invasion and metastasis, enabling replicative immortality (1, 2). Therefore, these driver genes have become the main candidates for targeted therapies in cancer treatments over the past decades. The most dramatic event in the journey of targeted therapeutics discovery is the identification of angiogenic targets (3). In 1971, Judah Folkman for the first time highlighted that angiogenesis was an important characteristic of solid tumors, which made anti-angiogenesis a potential therapeutic approach against various cancers (4, 5). Indeed, the anti-angiogenesis agents including vascular endothelial growth factor (VEGF) antibodies and receptor tyrosine kinases (RTK) inhibitors have been approved as first-line or second-line therapy for various solid cancers (6, 7). Later on, several types of molecular targeted therapies like apoptosis inducers and immunotherapies were developed and showed impressive curative benefits in treating patients with cancer (8, 9). Furthermore, other targeted therapies like HER2 based chimeric antigen receptor-T (CAR-T) and AMPK inhibitors are undergoing clinical evaluation for treating various cancers (10, 11).

However, cancer is a highly heterogenous disease with morphological diversity, distinct genetic alterations and microenvironmental discrepancies (12, 13). It would not be surprising that not all patients show responses to molecular targeted therapies. What is worse, many responsive patients become insensitive to the drugs after a certain period of treatment. Primary resistance or acquired resistance limit clinical benefits of targeted therapies to a large extent. Therefore, it is urgent to understand the underlying mechanism of therapeutic resistance. With the advent of new technologies such as CRISPR-Cas9 screening, and new experimental models like patient-derived xenograft (PDX) and patient-derived organoid (PDO), many advances have been achieved in decoding the mechanisms of drug resistance and identifying novel targets to predict or overcome drug resistance. Thus, this review summarized our recent understandings in resistance mechanisms, novel exploitable targets, and potential strategies to improve current modalities in molecular targeted therapies, in hope of shedding lights on future precision medicine for cancer patients.

## 2 Brief Description of Molecular Targeted Therapy

Although chemotherapy is the important therapeutic approach for cancer, its success is highly limited due to lack of selectivity, leading to insufficient elimination of tumor cells and systemic toxicity (14). Molecular targeted therapy is therefore gaining much attraction due to its specificity to cancer cells while sparing normal cells. In essence, molecular targeted therapies involve in developing drugs that block hallmarks of cancer (15). Therefore, identification of molecular targets represents major impetus for targeted cancer therapy *via* new technologies and approaches.

## 3 Major Classifications of Molecular Targeted Therapies

**Figure 1:**
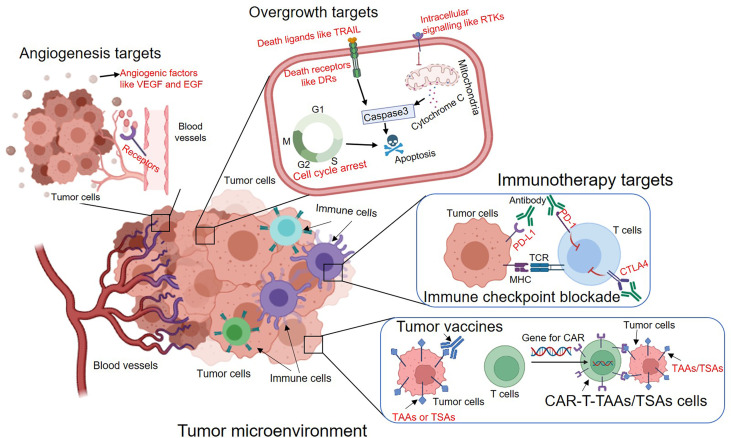
The overview of major types of molecular targeted therapies to inhibit tumour angiogenesis, overgrowth and immune evasion. The potential targets are indicated in red. VEGF, Vascular endothelial growth factor; EGF, Epidermal growth factor; TRAIL, TNF-related apoptosis-inducing ligand; DRs, Death receptors; RTKs, Receptor tyrosine kinases; PD-1, Programmed cell death protein 1; PD-L1, Programmed death-ligand 1; CTLA4, Cytotoxic T-Lymphocyte Associated Protein 4; TAAs, Tumor-associated antigens; TSAs, Tumor-specific antigens.

Since Hanahan et al. summarized essential hallmarks of cancer, including resisting apoptosis, sustaining proliferative signaling, inducing angiogenesis and evading host immunosurveillance. These typical properties provide the blueprint for exploring potential therapeutic targets. Indeed, most of molecularly targeted drugs were designed to interfere with cellular signaling pathways that fuel these properties. More importantly, these therapeutic strategies targeting hallmarks of cancer have been demonstrated to be effective in clinical settings. Thus, on the basis of these aggressive features of tumor cells, we summarize the following major classes of molecular targets for cancer therapy (**Figure 1**).

### 3.1 Overgrowth Related Molecular Targets

#### 3.1.1 Apoptosis Induction Related Targets

The cancer cell embodies features allowing it to survive beyond its normal life span *via* genetic mutations and epigenetics changes. Among them, aberrancies in programmed cell death and loss of growth control are critical steps in carcinogenesis. Although many signaling pathways mediate overgrowth of tumor cells, induction of cell apoptosis is a most typical way to inhibit cell overgrowth in molecular targeted cancer therapies (16). The discovery of the B cell lymphoma 2 (*BCL2*) gene in follicular lymphoma ignited interests in targeting this family to control cancer overgrowth by inducing cell apoptosis (17). Now the selective BCL2 inhibitors have been tested in clinical trials for patients with acute myeloid leukemia, non-Hodgkin lymphoma and multiple myeloma (18–20). In a phase I trial of Venetoclax (a BCL2 inhibitor), most patients with refractory chronic lymphocytic leukemia (CLL) had a partial response, while patients with chromosome 17p deletion achieved a complete response. Therefore, FDA approved Venetoclax in 2016 for the treating patients with 17p-deleted CLL that was refractory to at least one prior therapy (18). Later, Venetoclax was also approved as a combinatory therapeutic agent for patients with acute myeloid leukemia (AML) (21). The successful clinical translation of the first BCL2 inhibitor for treating haematological malignancies fostered development of other BCL family inhibitors. Several other BCL family inhibitors like ABT-737 and ABT-263 were developed to target both BCL2 and BCL-XL, respectively. Oltersdorf et al. reported that ABT-737 suppressed tumor growth in lymphoma and small-cell lung carcinoma as a monotherapy (22). Phase I trial further showed that 34.6% of patients with refractory CLL responded to ABT-263, and an overall response rate was 70% when it was combined with Rituximab (A chimeric monoclonal antibody against the protein CD20) in previously untreated patients (23, 24). In addition, other inhibitors targeting BCL family are also being evaluated in preclinical and clinical studies (25–27).

Later on, the second apoptosis pathway is also explored to design potential targeted therapies. This pathway includes Fas and the tumor necrosis factor (TNF) family related members like TNFRs and TNF-related apoptosis-inducing ligand (TRAIL) (28, 29). Tremendous work has focused on targeting TRAIL death receptors (DRs) through administration of TRAIL or agonist antibodies to the TRAIL DRs. ONC201, a first-in-class orally active TRAIL-inducing agent, displays immunostimulatory effects and triggers tumor cell apoptosis in both preclinical and clinical studies (30, 31). ABBV-621, another TRAIL agonist, is being investigated in a combinatory chemotherapy regimen for patients with colorectal cancer (CRC) or pancreatic cancer. Additionally, ABBV-621, as a monotherapy or in combination with Venetoclax, was also tested in patients with AML or diffuse large B cell lymphoma (DLBCL) (32, 33). Similarly, DR4 and DR5 agonists showed selectivity towards cancer cells without damaging to normal tissues in preclinical models (34). The DR5 activating antibody (Lexatumumab) has been investigated as a single agent or combined with chemotherapy in patients with osteosarcoma and Hodgkin lymphoma (35, 36). These studies have reinvigorated the interest in TRAIL-based cancer therapeutic strategies *via* apoptosis induction.

The success of abovementioned apoptotic related targets in oncology promotes researchers to explore other pathways involved in modulating overgrowth of tumor cells. Mitogen-activated protein kinase (MAPK) signaling pathway functions as one of most potent oncogenic pathways, in which many proteins have been recognized as potential targets regulating both programmed cell death and cell proliferation in tumor cells [reviewed in (37, 38)]. MAPK pathway is traditionally classified into mitogen and stress activated MAPKs. Normally, extracellular signaling-regulated kinase (ERK) works as mitogen responsive MAPKs while Jun N-terminal kinases (JNK) and p38 functions as stress responsive MAPKs. A wide variety of studies have shown that the dysfunction of RAS-RAF-MEK-ERK pathway is a major trigger for the development of cancer types (39). Not surprisingly, this pathway is one of hottest targets in cancer therapies. A great breakthrough was the approval of the B-Raf Serine/Threonine Kinase (BRAF) inhibitor named Vemurafenib in treating BRAF-mutated melanoma in 2002. In contrast, different clinical trials showed that BRAF inhibitor is less efficient in patients with papillary thyroid cancers and CRC (40, 41). This, at least in part, ascribes to the fact that melanoma shows high *BRAF* mutation (50–70% of cases) (42). Several additional BRAF inhibitors have, or are being, studied in phase I/II clinical trials (43). MEK inhibitors showed clear therapeutic responses in different clinical trials and were subsequently approved by FDA in patients with melanoma (44). Similarly, ERK1/2 and KRAS inhibitors also promote apoptosis and suppress proliferation, which are going to the clinical trials (45, 46). Furthermore, RTKs play important roles in initiating singling of MAPK pathway. It would not be surprising that RTKs are recognized as ideal candidates for cancer therapies. Various studies demonstrated that the impressive anti-cancer effects could be achieved by inhibition of RTKs such as human epidermal growth factor receptor 2 (HER2), mesenchymal epithelial transition factor (c-MET), insulin-like growth factor 1 receptor (IGF1R) and many others (47–50). These results were further confirmed in the various clinical trials (51, 52). Based on these promising clinical results, FDA approved several RTKs (HER2 and MET) inhibitors in treating human cancers (53).

PI3K/AKT/mTOR signaling cascade is another important oncogenic pathway in mediating both programmed cell death and cell proliferation. Phosphoinositide-3-kinase (PI3K) comprises various isoforms including PI3Kα, β, δ and γ. Although PI3Kα is most frequently dysregulated PI3K in cancers, other isoforms also contribute to tumorigenesis at various degrees. Therefore, different isoform-specific inhibitors have been developed and FDA approved some of them for molecular targeted cancer therapies. For example, Idelalisib (a PI3Kδ inhibitor) was approved in 2004 for relapsed or refractory chronic lymphocytic leukemia (CLL) in combination with rituximab in some patients (54). Similarly, Copanlisib (PI3Kα and PI3Kδ isoforms inhibitor) was approved by FDA in 2017 for the treatment of adult patients with relapsed follicular lymphoma who have received at least two prior systemic therapies (54). AKT is a major downstream effector in the PI3K signaling pathway and therefore is considered as an important therapeutic target. A variety of pre-clinical studies revealed that the AKT inhibitors like MK-2206 are the potential drugs against tumors with loss of phosphatase and tensin homolog (PTEN) function or PIK3CA mutations (55). In the clinic, most of AKT inhibitors are used as an adjunctive therapeutic agent, as AKT inhibitors alone displayed a limited clinical effect. Mammalian target of rapamycin (mTOR) is another important effector in PI3K pathway and plays critical roles in cancer cell growth (56). In addition, plenty of preclinical studies support that mTOR is an important therapeutic target in cancer (57). Indeed, mTOR inhibitors were approved for the treatment of human cancers including advanced neuroendocrine tumors and advanced breast cancer (58). Furthermore, Temsirolimus (a mTOR inhibitor) was approved for the treatment of advanced stages of renal cell carcinomas (59). Other mTOR inhibitors are currently under clinical trials. In conclusion, the oncogenic pathways including PI3K/Akt/mTOR signaling pathway represent a class of candidates in targeted cancer therapies due to their important oncogenic functions in regulating cell apoptosis.

In addition, several different types of targets have been identified to mediate apoptotic processes, including Poly (ADP-ribose) polymerases (PARPs), epigenetic and cellular stress regulators. PARPs are abundant nuclear protein activated by DNA breaks capable of synthesizing poly (ADP-ribose) (PAR) chains that serve as signals for the recruitment of several DNA repair proteins. When excessive DNA damage occurs, PARP-1 can regulate apoptosis in cancer cells by changing the activity and localization of cytoplasmic proteins like apoptosis-inducing factor (60, 61). Therefore, PARPs have been considered as potential targets in treating cancer, especially when cancer cells harbor BRCA1/2 mutations. These BRCA1/2 mutations impair homologous recombination function of cancer cells, which lead to accumulation of DNA double strand breaks caused by inhibition of PARPs (62). Indeed, a series of studies showed that inhibition of PARPs provided a significant therapeutic benefit in cancer (63–66). These promising results eventually result in approval of four PARP inhibitors (Olaparib, Rucaparib, Niraparib and Talazoparib) in treating different types of cancer in particular with BRCA mutations (67). Recently, epigenetic approaches have been studied in mediating apoptosis induction. For example, inhibitors of histone deacetylases primed rhabdomyosarcoma to Venetoclax-induced apoptosis by increasing the expression of BCL2L11 (68). In multiple myeloma cells, histone deacetylases inhibitors also enhanced the activity of mitogen-activated protein kinase (MEK) inhibitors and Venetoclax (69, 70), which is being evaluated in the phase I clinical trials. In addition, there are also ongoing efforts to increase cellular stress, thus inducing cancer cell apoptosis. Multiple chaperone proteins like heat-shock family and endoplasmic reticulum stress members have been studied in their ability in apoptosis induction. Preclinical studies showed that the heat shock protein 90 inhibitors induced apoptosis in cancer cells (69, 71). Altogether, these new identified targets expand repertoire of apoptosis related modulators, providing better options to cure cancer.

#### 3.1.2 Proliferative Signaling Related Targets

Cell cycle regulatory proteins directly control growth of tumor cells *via* G0/G1, S, G2 and M phases. It is thus reasonable that cell cycle proteins like cyclin-dependent kinases (CDKs) family could be potential targets in cancer treatment. Although Pan-CDKs inhibitors like Flavopiridol and Dinaciclib blocked tumor cell proliferation in xenograft models of human cancers including ovarian and pancreatic carcinoma, they showed little clinical activity (72–78). This led to the development of CDK-selective compounds including Palbociclib, Ribociclib and Abemaciclib (79), which have been showed strong antitumor activity in various malignancies like glioblastoma and CRC (80–82), leading to their widespread evaluation in more than 30 clinical trials (83). Of note, 23% of patients with metastatic breast cancer showed partial responses to Abemaciclib as a monotherapy. Due to its potent anticancer activity, Abemaciclib was approved for treating HR and HER2 positive breast cancer by the FDA. Beside CDKs, inhibitors targeting other cell cycle proteins like checkpoint kinase 1 (CHK1) and G2 checkpoint kinase (WEE1) have been developed and assessed in preclinical and clinical studies (84–87). Similarly, the clinical benefit of WEE1 inhibitors was under clinical evaluation (88, 89). Overall, these studies suggest that inducing apoptotic process and promoting mitotic arrest are effective approaches in treating human cancers.

### 3.2 Angiogenesis Related Molecular Targets

Angiogenesis is an essential event in metastatic dissemination of cancer cells. When the transformed progenitor cells grow to a certain size, tumor cells need sufficient supply of oxygen and nutrients, initiating angiogenesis to support their malignant growth (90). Therefore, inhibition of new blood vessel formation is an ideal approach to treat solid tumors, in particular their metastases. So far, tremendous attempts have been undertaken to establish anti-angiogenics as rational components for improving the unmet clinical challenges in cancer treatment. One of key findings is the discovery of VEGF family and its receptors (91). Various studies have led to identification of several exploitable angiogenesis targets like VEGFA and VEGFR2. In 2003, the FDA approved Bevacizumab, a humanized VEGF neutralizing monoclonal antibody, as the first anti-angiogenic agent for combinatorial treatment of metastatic CRC and subsequently for treating patients with non-small-cell lung cancer (NSCLC) (92, 93). Since then, several VEGF inhibitors blocking the VEGF-VEGFR axis are being tested in different phases of clinical trials (94).

Besides VEGF family, tumor angiogenesis is also tightly regulated by numerous endogenous factors like epidermal growth factor receptor (EGFR) and fibroblast growth factor (FGF). Activation of the EGF-EGFR signaling pathway enhances the formation of new blood vessels in tumor tissues. Blockade of the EGF-EGFR signaling shows promising curative responses in patients with different cancers (95, 96). The combination of Cetuximab (an EGFR inhibitor) with chemotherapy led to significant improvement in overall survival in patients with CRC, compared to chemotherapy alone (97). Consistently, higher progression free survival was also observed in CRC patients treated with Cetuximab together with chemotherapy (98). Therefore, Cetuximab was approved to treat patients with NSCLC and CRC (99).

In addition, tumor angiogenesis is controlled by several other signaling pathways, which could also be potential targets for anti-angiogenic therapy. For instance, Maxwell et al. reported that absence of hypoxia inducible factor-1β (HIF-1β) in tumor cells gave rise to poorly vascularized tumors with reduced VEGF expression (100). Consistently, deletion of HIF2 in murine endothelial cells lowered the sensitivity of tumor cells to hypoxic stress and led to defective tumor angiogenesis (101). Thus, beneficial outcome of targeting hypoxia regulatory pathway has been tested in several clinical trials (102, 103). Interestingly, axon-guidance molecules like ephrins and their Eph receptors have also been reported as important roles in tumor angiogenesis. The ephrin A1 mediates TNF-α-induced angiogenesis *in vivo* (104). Interfering with EphA signaling resulted in impaired tumor angiogenesis (105). In addition, some other cytokines and growth factors could be potential targets in antiangiogenic therapy (106, 107). Altogether, tumor angiogenesis-regulatory signaling pathways represent important targets in targeted cancer therapies.

### 3.3 Immunotherapy Targets

#### 3.3.1 Immune Checkpoint Related Molecular Targets

Tissue homeostasis is maintained by host immunosurveillance *via* cytotoxic innate and adaptive immune cells, but the transformed cells usually escape tumoricidal immune clearance *via* multiple resistance mechanisms. One of them is that tumor cells mimic peripheral immune tolerance *via* immunosuppression network. Cytotoxic T lymphocyte antigen 4 (CTLA4) and programmed cell death 1 (PD-1) are most known negative regulators of T-cell immune function (108). Inhibition of these targets leads to increased activity of the immune system. Preclinical studies showed that blockade of CTLA4 or PD-1 repressed tumor growth, which provide pioneering evidence for treating cancer patients with immune checkpoint inhibitors (109, 110). A series of clinical trials later demonstrated that inhibition of CTLA4 or PD-1 led to desirable responses and improved overall survival in patients with various cancers including melanoma and squamous-cell non-small-cell lung cancer (111–113). Due to these promising clinical studies, monoclonal antibodies against CTLA4 or PD-1 were eventually approved for treating various cancers including melanoma, lung cancer, kidney cancer and breast cancer (114). More clinical studies involving CTLA4 or PD-1 blockade are being evaluated for other types of cancer including mesothelioma, sarcoma and CRC (115–119). Similar to PD-1, the blockade of the PD-1 ligand (PD-L1) is also beneficial to enhance host antitumor immunosurveillance. Indeed, PD-L1 antibodies have been proven effective in treating multiple human cancers. Therefore, the humanized PD-L1 monoclonal antibodies including Atezolizumab, Avelumab and Durvalumab were approved for treatment of human cancers such as urothelial carcinoma and renal cell carcinoma (120).

The success of CTLA4 and PD-1/PD-L1 blockade in cancer treatment results in the discovery of novel inhibitory regulators of T cell activation, including Lymphocyte activation gene 3 (LAG3), T cell immunoglobulin 3 (TIM3), V-domain immunoglobulin suppressor of T cell activation (VISTA) and B7-H3 (121). Preclinical studies have reported that inhibition of these immune checkpoints elicited potent antitumor effects in different types of cancer. For instance, anti-VISTA antibody prolonged the survival of tumor-bearing mice by promoting T cell proliferation and cytokine production (122). Furthermore, clinical trials are being investigated to evaluate the efficacy of blocking these inhibitory regulators (123–125).

#### 3.3.2 Tumor Vaccine Related Targets

Unlike preventive vaccines, tumor vaccines are a therapeutic strategy aimed at eliciting a specific *in vivo* immune response against tumor antigens. Therefore, tumor associated antigens (TAAs) are important for development of tumor vaccines. Many tumor-associated antigens (TAAs) have been identified, some of which are shared with normal tissues, whereas others are specific to tumors. TAAs are derived from the aberrantly overexpressed self-antigens like cancer–testis antigens and MUC-1 in tumor cells compared to normal cells (126, 127). The overexpression of these TAAs is able to induce an antitumor immune response when expression level of these proteins reaches the threshold for T cell recognition, thereby breaking immunological tolerance. Several therapeutic tumor vaccines based on TAAs have been tested for distinct human cancer in different phases of clinical trials (128, 129). For example, Stimuvax (BLP25 liposome vaccine) targeting MUC1 for NSCLC is in the phase III trial. However, these tumor vaccines show limited efficacy. For instance, the trial evaluating the efficacy of Nelipepimut-S antigen in preventing breast cancer recurrence showed that no significant between-arms differences in disease-free survival events at the median follow-up (16.8 months) (130). This could be ascribed to multiple reasons including the low affinity between these antigens and T cell receptors and the tumor evasion with loss of tumor antigen expression. In addition, TAAs are subject to some degree of central tolerance and lack complete specificity to the tumor, which also limit their clinical benefit. These limitations obtained with cancer vaccines based on TAAs urged the development of new strategies, in particular, the identification of specific antigens. Tumor-specific antigens (TSAs) are strictly specific to tumors, which often arise from mutated neoantigens (131, 132). So far, feasibility and immunogenicity of tumor vaccine based on TSAs were confirmed in several clinical trials (133). In particular, patients with melanoma treated with patient-specific mutated neoantigens responded to vaccination. Vaccinated patients showed efficiently delayed tumor recurrence with the expansion of the repertoire of neoantigen-specific T cells (134, 135). These promising results indicate that tumor vaccine based on personalized neoantigen opens a new approach for cancer targeted immunotherapy.

#### 3.3.3 Chimeric Antigen Receptor T-Cell Related Targets

Chimeric antigen receptor (CAR) T-cell therapy represents a novel immunotherapy in cancer treatment. In this strategy, a patient’s own T cells are genetically engineered to express a synthetic receptor that can specifically interact with the tumor-associated antigens (TAAs) expressing on tumor cell surface. The pioneering trails of CAR-T-CD19 therapy in patients with lymphoma or leukemia, resulting in FDA approval of two distinct anti-CD19 CAR T cell products for the treatment of both acute lymphoblastic leukaemia and diffuse large B cell lymphoma (136–138). Since then, various TAAs could act as target antigens for CAR-T cells. For example, the type III variant EGFR (EGFRvIII) was considered as an ideal target for its aberrant expression on the cell surface of cancer cells (139). Importantly, the engineered T cells expressing CAR can specifically recognize EGFRvIII cell lines, whilst no reactivity to co-cultured normal tissue cells. Currently, CAR-T-EGFRvIII cells have been tested in patient with glioblastoma (140). Likewise, the CAR-T- HER2 cells also showed encouraging results in antitumoral effects in different types of cancer including osteosarcoma and medulloblastoma (141, 142). These promising results led to the clinical trial of CAR-T- HER2 cells in patients with sarcoma (10). Still, CAR-T-TAAs can be further modified in order to enhance the antitumor efficacy. For example, Brentjens et al. developed T cells co-expressing MUC16 (a well-known ovarian tumor antigen) CAR and IL-12, and IL-12 secreting CAR-T-MUC16 cells exhibit enhanced antitumor efficacy as determined by increased survival, prolonged persistence of T cells (143). Based on this rationale, they initiated a phase I clinical trial in patients with recurrent ovarian cancer (144). Chen et al. also reported that dual-targeted CAR-T cells co-expressing glypican-3 (GPC3) and asialoglycoprotein receptor 1 (ASGR1) exerted superior anticancer activity and persistence against single-targeted T cells in two GPC3^+^ASGR1^+^ hepatocellular carcinoma (HCC) xenograft models (145). Interestingly, they also found that no obvious growth suppression was seen with single or double antigen-negative HCC xenografts, indicating that dual-targeted CAR-T cells is a potential way to reduce on-target, off-tumor toxicity. With the development of next generation of sequencing, more cancer cell specific TAAs have been identified, which extensively improve the designation of tailored CAR-T-TAAs cells for cancer patients.

## 4 The Mechanisms of Resistance to Molecular Targeted Therapies

Clinical application of molecular targeted drugs has led to significant improvement in the survival and quality of life of patients. However, drug resistance represents a major obstacle to limit sustained clinical benefits of these targeted cancer therapies. Most cancer patients do not respond to molecular targeted drugs due to primary resistance. Alternatively, some responders eventually suffer from cancer relapse after a period of response, resulting from acquired resistance. Mutations or low expression of the targets or inactivation of targets related signaling pathways are the potential mechanisms of primary resistance (146, 147). For instance, the *de novo MET* amplification caused primary resistance to EGFR inhibitors in *EGFR*-mutant NSCLC (148). In contrast, acquired resistance could be ascribed to multiple reasons including activation of bypass pathways, alterations in the therapeutic targets themself and adaptive survival mechanisms (147). For example, in chronic myeloid leukemia the activation of GCA-TRAF6-ULK1 autophagy regulatory axis was associated with the acquired resistance to Imatinib (a tyrosine kinase inhibitor) (149). Therefore, it is still urgent to explore novel therapeutic opportunities to overcome drug resistance. In this regard, recent advances in cutting-edge methodologies including CRISPR-Cas9 screening and patient-derived models significantly accelerated our research in exploring the unappreciated mechanisms of resistance to molecular targeted drugs.

### 4.1 The Application of CRISPR-Cas9 Screening in Unraveling the Mechanisms of Resistance to Targeted Therapies

#### 4.1.1 Classification of CRISPR-Cas9 Screening

**Figure 2:**
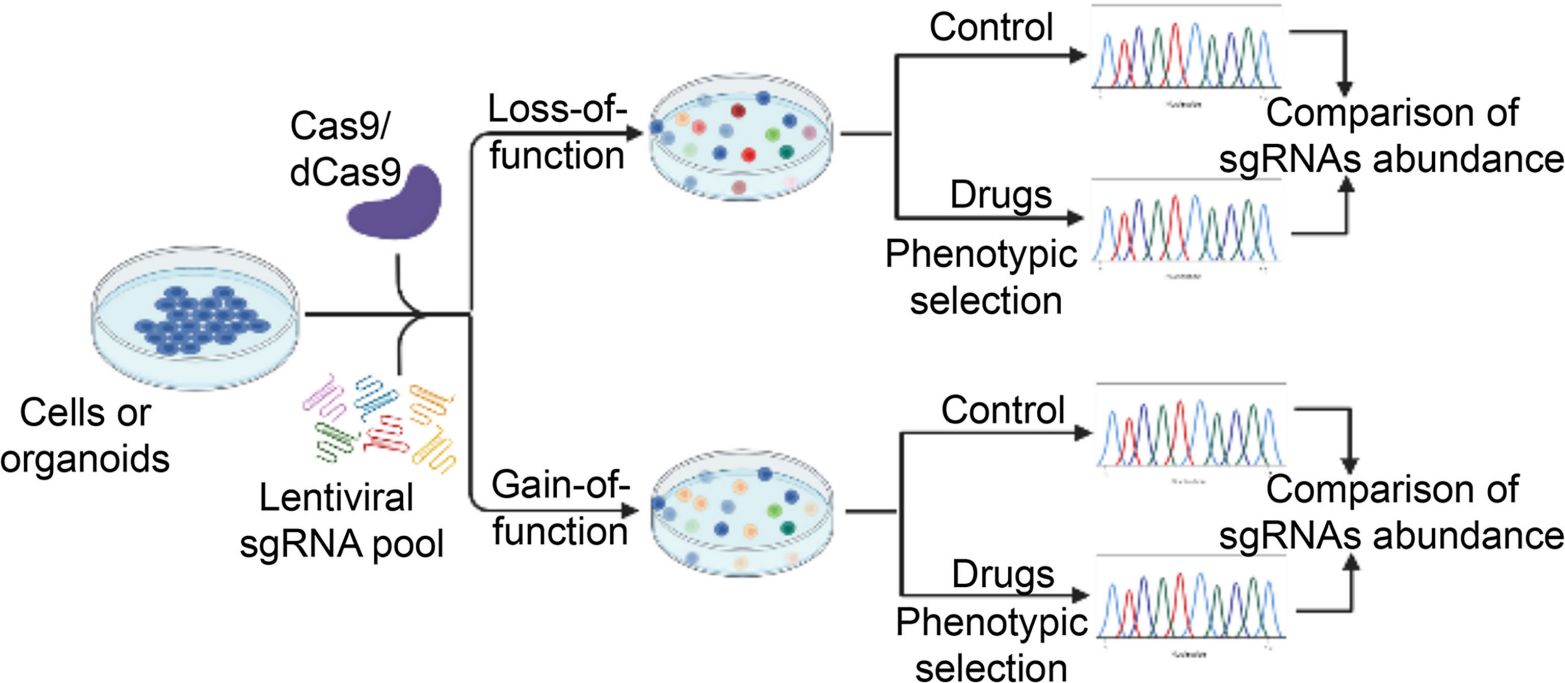
A schematic diagram illustrates the workflow of high-throughput CRISPR-Cas9 screening for novel regulators of drug sensitivity. CRISPR-Cas9 sgRNA libraries are packed into lentiviral vectors and transfected into Cas9- or dCas9-expressing cancer cells. In the case of loss-of-function screening, CRISPR-Cas9-mediated genome editing leads to gene knockout (or transcriptional inhibition) in individual cells, which are subsequently selection by various drugs. The residual drug-resistant cells are collected. The abundance of cells with different sgRNAs is determined in the drug-treated and control pool. Cells with sgRNAs targeting genes that cause drug resistance upon knockout (or transcriptional inhibition) will be enriched while those resulting in enhanced sensitivity to the drug will be depleted in the final pool. For gain-of-function screening, activation of gene expression by dCas9-mediated recruitment of transcriptional activation domains to transcriptional start site. Other procedures are similar to loss-of-function screening. The unique sgRNA sequence in the genome serves as a genetic barcode for high-throughput phenotyping by next-generation sequencing. Essential genes for drug sensitivity are identified for further validation. dCas9, nuclease-dead Cas9. sgRNA, single guide RNA.

CRISPR-Cas9 gene editing technology can target components of the whole genome containing the promoters, enhancers, introns and inter-genic regions, thus CRISPR-Cas9 technology has become a powerful tool for completely eradicating the target gene (150). In addition, accumulating studies now also utilize the catalytically-dead mutant of Cas9, referred to as dCas9, in which the nuclease activity of the Cas9 has been lost. dCas9 has been fused to an array of chromatin modifiers to convert it into a highly versatile enzyme that can be used to perform activation (CRISPR-dCas9 activation, CRISPRa) or repression (CRISPR-dCas9 interference, CRISPRi) screening (151). Therefore, CRISPR-Cas9 technology can provide the opportunity to study the mechanism of drug resistance and contribute to identification of several resistance-related genes by the large-scale screening (CRISPR-Cas9 screening). The modes of CRISPR-Cas9 screening can be divided into two forms: 1) loss of function screen, 2) gain of function screen. The former can be done by CRISPR-Cas9 knockout and CRISPRi, while the latter is achieved by CRISPRa. Furthermore, loss of function screen can be further separated into negative and positive selection screen on the basis of different purposes (**Figure 2**). The purpose of a negative selection screen is to identify perturbations that affect the survival or proliferation of cells, which cause the perturbed cells to be depleted during selection. Such screens have been widely used to identify both essential genes that are required for cell lines tested and a small set of genetic dependencies of specific cancer cell lines (152–154). In contrast, a strong selective pressure is necessary for positive selection screen, so that the probability of cells being selected without the genetic perturbation is low. Therefore, positive screen is important for identifying perturbations that confer resistance to drugs.

Below we will further outline the application of CRISPR-Cas9 screening in exploring the mechanisms of resistance to molecular targeted therapies and discovering novel targets in various tumors, according to the classification of hallmarks of cancer (**Table 1**).

**Table 1:** Major types of molecular targeted therapies in which CRISPR-Cas9 screening was applied to explore the mechanisms of drug resistance and novel therapeutic targets.

Major types of molecular targets	Molecularly targeted drugs	The cancer types	Newly identified oncogenic Targets	Newly identified Tumor suppressive Targets
Anti-overgrowth related molecular targets	EGFR inhibitor	*EGFR*-dependent non-small cell lung cancer	*Capicua* (155)	
		*NRAS/KRAS/BRAF^V600^*-wild type colorectal cancer		*NF1* (191)
	FGFR inhibitor	Gastric cancer	*ILK* (157)	*CSK* (157)
	PI3K inhibitor	Pancreatic cancer	*MEMO1, YPEL5* (191)	*NF1* (158)
	MEK inhibitor	*RAS*-mutant melanoma		*FBXO42* (160)
		*KRAS*-mutant Pancreatic and lung cancers	*SHOC* (190)	
		Lung cancer	*MAPK7* (158)	
	MAPK inhibtor	*BRAF^V600^*-mutant melanoma		*SIRT6* (173)
	CDK4/6 inhibitor	Bladder cancer	*KDR, FGFR3, AKT3, JAK2, STAT3* (196)	
	BCL2 inhibitor	Acute myeloid leukemia	*CLPB* (165)*, RBFA* (162)	
	PARP inhibitor	Breast cancer	*RNASEH2B* (168)	*C20orf196, FAM35A, PARP1* (166)
	PARP inhibitor	Ovarian cancer	*C12orf5* (193)	
	HDAC inhibitor	Multiple myeloma	*ABCB1* (169)	
	MEK inhibitor and CDK4/6 inhibitors	*NRAS*-mutant melanoma	*KRAS* (171)	
	MEK inhibitor and CDK7/12 inhibitor	*EGFR*-dependent lung cancer	*EP300, CREBBP, MED1* (197)	
	HER2 inhibitor	Breast cancer	*TALDO1* (172)	
	Multiple inhibition of EGFR, ALK, BRAF, MEK	Lung cancer cell with *EGFR, ALK, BRAF, KRAS*, or *NRAS* mutations		*KEAP1* (192)
Anti-angiogenesis related molecular targets	VEGFR/PDGF inhibitor	Hepatocellular carcinoma	*PHGDH* (201), *CDK12* (182)	*SGOL1* (181)*, KEAP1* (183), *LATS2* (202)
	EGFR inhibitor	Non-small cell lung cancer	Tankyrase (177), *RIC8A* (178)	*ARIH2* (178)
		Glioblastoma	PJA1 (179)	
		EGFR-mutant lung cancer	*TBK1, TRIB2* (180)	
Immune checkpoint related molecular targets	Anti-PD1/PD-L1/ CTLA4 antibodies	Melanoma	*Ptpn2, H2-T23* (184), *ADAR1* (185), *JAK1* (186),	*APLNR* (186)
		Pancreatic cancer	*CMTM6* (205)	
		*ALK*+ anaplastic large-cell cancer	*GRB2/SOS1, IRF4, BATF* (185)	
		Ovarian cancer	*EGFR* (186)	
		Lung cancer	*ASF1A* (188)	
CAR-T-cell related targets	CAR-BCMA-T cells	Multiple myeloma	*HDAC7 and* *Sec61* (213)	

#### 4.1.2 The Mechanisms of Resistance to Apoptosis Inducing Targeted Therapies

Although several drugs have been successful developed in treating cancer by interfering with MAPK/ERK and PI3K/AKT/mTOR pathways, drug resistance often occurs and limits their durable clinical benefit. For instance, EGFR is a key RTK that initiates MAPK/ERK and PI3K/AKT/mTOR pathways. Even though EGFR-targeted cancer therapy is initially effective against *KRAS*-wild-type cancers, drug resistance is prevalent in patients receiving several cycles of treatment, highlighting the urgent need to design novel therapeutic modalities. Based on CRISPR-Cas9 screening, Liao et al. reported that Capicua, a transcription repressor, restricted the efficacy of EGFR inhibitors through regulating EGFR expression (155). Thus, *Capicua* mutations suppressed the effect of EGFR inhibitor in the EGFR-dependent NSCLC cells. In a genome-scale CRISPR-Cas9 modifier screening, neurofibromin 1 (NF1) was identified as an important regulator of the resistance to EGFR inhibitors in *NRAS/KRAS/BRAF^V600^*-wildtype CRC cells (156). Mechanistically, NF1 deficiency leads to sustained activation of the MAPK pathway signaling to promote cell proliferation in the presence of EGFR inhibitors. Therefore, MEK inhibitor and EGFR inhibitor displays synergistic antitumor activity in these settings (156). By applying domain-based CRISPR-Cas9 screening, Wang et al. identified that depletion of tankyrase or its associated E3 ligase enhanced the growth inhibitory activity of EGFR inhibitor in NSCLC through stabilizing angiomotins to inhibit YAP signaling (177). Using similar strategy, they further found that knockout of RIC8 guanine nucleotide exchange factor A, a positive regulator of YAP signaling, sensitized *EGFR*-mutant NSCLC to EGFR inhibitor. In contrast, knockout of ARIH2, a component of the Cullin-5 E3 ubiquitin ligase complex, induced the resistance to EGFR inhibitor. In keeping with this finding, inhibition of E3 ubiquitin ligase PJA1 increased efficacy of EGFR blockade by stabilizing the downstream effector Capicua (178, 179). Moreover, a genome-wide loss of function CRISPR-Cas9 screening in *EGFR*-mutant lung cancer cells identified several EGFR-dependent essential genes including TANK binding kinase 1 and Tribbles pseudokinase 2. Knockout of these essential genes significantly decreased tumorigenic properties of EGFR-mutant lung cancer cells (180). These newly identified resistance regulators *via* high throughput CRISPR-Cas9 screening provide not only biomarkers to predict clinical response, but also potential molecular targets for the combination therapy.

Except for anti-EGFR related resistance, the potential mechanisms of resistance to other targets in MAPK/ERK and PI3K/AKT/mTOR pathways have been unraveled. For example, as a receptor of FGF, main function of FGFR, a member of RTKs family, is to amplify the FGF signal transduction to RAS-ERK and PI3K-AKT signal cascade. Through a CRISPR screen implemented in gastric cancer cell line, ILK, CSK and EGFR pathways have been identified as critical roles in the resistance to FGFR inhibitor. ILK and EGFR are both the significantly depeleted genes in this screen, which means the combinational inhibition of them and FGFR can ideally boost the efficacy of the treatment to gastric cancer (157). Meanwhile, ERN1-JNK-JUN pathway was identified as a bypass cell signaling that mediates resistance to MEK inhibitor in *KRAS* mutant CRC cells through a synthetic lethal CRISPR-Cas9 screening. Consistently, compounds targeting JNK/MAPK8 or TAK1/MAP3K7, which relay signals from ERN1 to JUN, displayed synergy with MEK inhibition (189). Moreover, loss of both *Capicua* and *FBXO42* reduced sensitivity to MEK inhibition in *RAS*-mutant cancers *via* different mechanisms (159, 160). In another study of CRISPR-Cas9 screening, suppression of SHOC, a positive regulator of MAPK signaling, sensitized *KRAS*-mutant pancreatic and lung cancer cells to MEK inhibitors (190). Yau et al. performed the CRISPR-Cas9 screening in human *KRAS* mutant CRC cells, and found that genetic or pharmacologic disruption of the metabolic enzymes nicotinamide adenine dinucleotide kinase (NADK) or ketohexokinase inhibited tumor growth *in vivo*, indicating that NADK inhibitors can be promising candidates for combinational therapy (161). By using the similar approach, inhibition of MAPK7, an essential MEK gene, attenuated the re-activation of MAPK signaling following long-term MEK inhibition in *KRAS* mutant cancer cells, suggesting that targeting MAPK7 may attenuate acquired resistance to MEK inhibition (158). Combinational therapy using MAPK7 inhibitors and MEK inhibitors may improve therapeutic efficiency especially for lung cancer patients. Based on CRISPR-Cas9 screening, ERBB and mTOR signaling network were found to be key determinants of response to PI3K inhibition. Knockout of unexplored genes like *MEMO1* and *YPEL5* in these signaling networks sensitized cancer cells to PI3K inhibitors (191). These studies reveal that combinatory targeting of these drivers may overcome drug resistance in clinical settings.

In addition, mitochondria-associated genes were identified to be essential for survival of *KRAS*-mutant cancer cells. It would not be surprising that mitochondrial inhibitors reduced the growth of *KRAS* mutant cancers (164). Chen et al. identified that CLPB, an ATPase regulator, was increased upon acquisition of Venetoclax (a BCL2 inhibitor) resistance in human AML by applying CRISPR-Cas9 screen (165). Mechanistically, CLPB maintained the mitochondrial cristae structure *via* its interaction with the cristae-shaping protein OPA1, whereas its loss promoted apoptosis by inducing cristae remodeling and mitochondrial stress responses. Consistently, high expression of the mitochondrial translation machinery genes like *RBFA* contributed to resistance to Venetoclax in AML, indicating that targeting mitochondrial translation machinery may be a promising approach to circumvent Venetoclax resistance (162).

In *BRAF^V600^* mutant cancer cells, the haploinsufficiency of histone deacetylase Sirtuinb 6 (SIRT6) allowed *BRAF^V600^*-mutant melanoma cell persistence in the presence of MAPK inhibitors due to upregulating insulin like growth factor binding protein 2 (IGFBP2) expression *via* increasing chromatin accessibility at the IGFBP2 locus (173). Therefore, resistance to BRAF inhibitors in SIRT6-haploinsufficient melanoma cells can be relieved by combinatory application of a clinically available IGF-1R inhibitor. These results suggest that combinatory targeting these newly identified drivers may overcome drug resistance in cancer cells bearing *KRAS* and *BRAF* mutations. Similarly, in lung cancer cells with mutant *EGFR* and *BRAF*, Krall et al. also reported that loss of *KEAP1* (Kelch-like ECH associated protein 1) diminished the therapeutic effects of EGFR, BRAF and MEK inhibitors, indicating that *KEAP1* is a biomarker to predict the responses of these inhibitors (192).

PARP inhibitors also induce apoptosis processes and are standard molecular therapeutic drugs for BRCA1/2-defecient cancers (167). However, drug resistance is observed in subset of patients upon widespread clinical applications. To dissect potential underlying mechanisms, Dev et al. performed whole-genome CRISPR-Cas9 synthetic-viability/resistance screening in BRCA1-deficient breast cancer cells treated with PARP inhibitors and identified that the resistance of breast cancer cells to PARP inhibitor was, at least in part, due to loss of *C20orf196* and *FAM35A* (166). This finding can aid patient stratification and yield new treatment opportunities. In contrast, mutation of *RNASEH2B* which encodes ribonuclease H2, sensitize tumor cells to PARP inhibition (168). CRISPR-Cas9 screening also identified glycolysis and apoptosis regulator (TIGAR), also known as *C12orf5*, as a regulator of PARP inhibitors responsiveness in ovarian cancer cells. Indeed, knockdown of TIGAR promoted the sensitivity to the Olaparib (a PARP inhibitor) (193). All in all, CRISPR-Cas9 screening has provided a powerful tool to elucidate mechanisms of drug resistance to inducing apoptosis, which not only contribute to discovering new targets but also aid the identification of novel biomarkers of therapeutic sensitivity.

#### 4.1.3 The Mechanisms of Resistance to Proliferative Signaling Targeted Therapies

Cell cycle progression related genes like CDKs have been considered as therapeutic targets by regulating proliferative signaling in various cancers, however, various clinical trials reported that monotherapy with CDK4/6 inhibitors has failed to provide long-term therapeutic effects in several tumor entities examined, largely due to primary and acquired resistance mechanisms (163, 194, 195). Using CRISPR-Cas9 gain of function screening, *KDR, FGFR3, AKT3, JAK2* and *STAT3* have been identified as key players mediating resistance to CDK4/6 inhibitors in bladder cancer cells (196). These results underscored the importance of combination therapy when using CDK4/6 inhibitors in clinical settings. Indeed, several combinatory therapeutic modalities have been postulated, including CDK4/6 inhibitors plus MK-2206 (a *AKT3* inhibitor), and CDK4/6 inhibitors plus Erdafitinib (a FGFR inhibitor) (170). Although combinatorial therapies can overcome the monotherapy resistance to some extent, emerging evidence shows that double targets-based combinatorial strategies also lead to resistance. For instance, in the case of NRAS mutant melanoma, Hayes et al. revealed that the resistance to combined therapy of MEK1/2 and CDK4/6 inhibitors was partial attributed to the activation of RTK-PI3K-AKT and RTK-RAS-RAF signaling pathways. In particular, activated KRAS was sufficient to confer resistance to combined MEK1/CDK inhibition (171). Another CRISPR-Cas9 screening study identified that expression of certain transcriptional regulators like *EP300* and *MED1* negatively correlated with inhibitory effect of Erlotinib/THZ1 (a CDK7 inhibitor) synergy in lung cancer cells (197). Therefore, the addition of *EP300* inhibitors to Erlotinib/THZ1 combinatorial therapy enhanced the synergistic effect of Erlotinib/THZ1 (198). Histone deacetylases (HDACs), a formation of post-translational modification, play a crucial role in biological functions, such as regulation of gene expression, chromatin dynamics, cell cycle progression, cytoskeletal dynamics, development events and autophagic processes. The mutation of gene encoded HDACs can induce the tumorigenesis. HDACs inhibitors (HDACi) have been approved for treating cancers, but resistance to HDACs inhibitors still exists (199, 200). Through CRISPR screen implementing in the multiple myeloma cells treated with panobinostat, an oral broad-spectrum HDACi, genes encoding for the cell surface ABC transporters ABCB1 (MDR1/p-glycoprotein) have been identified as the most prominently reason for resistance to HDACi. With the combination of HDACi, Elacridar, a third-generation inhibitor of ABCB1, is a promising candidate to promote the efficacy of HDACi (169). Majority of targeted therapy in breast cancer and ovarian cancer focus on HER-2, through CRISPR/Cas9 loss of function screen, Xiao-Fan Wan manifests that TALDO1 is critical for the survival of breast cancer cell line with the treatment of HER2 inhibitor (172). These key regulators identified by CRISPR-Cas9 screening might provide potential target to design triple combinatory strategies to circumvent drug resistance to proliferative signaling targeted therapies.

#### 4.1.4 The Mechanisms of Resistance to Anti-Angiogenesis Therapies

VEGF is a potent growth factor that promotes the blood vessel formation in tumor tissues, so most anti-angiogenesis therapies are designed to block the VEGF-VEGFR signaling axis. However, various studies showed that inhibition of VEGF signaling upregulated components of the bypass pathways such as fibroblast growth factors 2 (FGF2), angiopoietin (2ANGPT2) and HIF family members, which resulted in anti-angiogenesis resistance in tumor cells (174). For instance, Sorafenib (a multiple-kinase anti-angiogenesis inhibitor) treatment restrained tumor growth partly through suppression of tumor angiogenesis. However, tumor hypoxia associated with the Sorafenib treatment can induce the expression of VEGF and other proangiogenic factors that confer HCC resistance to Sorafenib treatment (175, 176). Wei et al. identified phosphoglycerate dehydrogenase (PHGDH) as a critical driver for Sorafenib resistance. Therefore, treatment of NCT-503 (a PHGDH inhibitor) acted synergistically with Sorafenib to suppress HCC growth *in vivo* (201). In another study, Shugoshin 1 (SGOL1) was identified as a prognostic indicator in patients treated with Sorafenib. Therefore, loss of SGOL1 reduced apoptosis upon Sorafenib treatment (181). Moreover, CRISPR-Cas9 screening also identified that inhibition of CDK12 was synergic with Sorafenib for HCC treatment (182). Using a similar CRISPR-Cas9 screening method, KEAP1 was identified as a regulator of resistance to Regorafenib (a VEGFR2 inhibitor) (183). Depletion of *KEAP1* also restored cell viability and lowered reactive oxygen species levels in cells incubated with Lenvatinib (a VEGFR2 inhibitor) (183). In addition, large tumor suppressor kinase 2 (LATS2) was found to regulate Regorafenib sensitivity in HCC cells, as LATS2 deletion stabilized the Yes-associated protein (YAP) to upregulate antiapoptotic protein Bcl-xL and the multidrug resistance transporter ATP binding cassette subfamily B member 1. Consistently, knockdown of LATS2 significantly mitigated the cytotoxic and proapoptotic effects of Regorafenib on HCC cells (202). Furthermore, YAP activation might confer Regorafenib resistance in HCC cells through affecting tumor vasculature (202).

#### 4.1.5 The Mechanisms of Resistance to Immune Checkpoint Blockade

Although immune checkpoint blockade (ICB) including anti-PD-1/PD-L1 and anti-CTLA4 strategies induces long-lasting responses in cancer patients, the primary and acquired resistance largely limits its clinical application. Preliminary studies have found that activation of cell signaling pathways such as the interferon-gamma (IFNγ) pathway confers resistance to ICB, however the underlying mechanisms of unresponsiveness to ICB in various types of cancers are still poorly understood (203).

To approach this issue, Manguso et al. developed *in vivo* CRISPR-Cas9 screening to identify specific genes regulating the sensitivity to the immune checkpoint inhibitors (184). Mice were injected with B16 melanoma cells engineered with the Cas9 gene and an sgRNA library, and then treated by PD-1 antibodies. High throughput sequencing uncovered that protein tyrosine phosphatase non-receptor type 2 (*PTPN2*) *and H2-T23* were novel immune-suppressing molecules dictating the efficacies of ICB. They also demonstrated that the efficacy of immunotherapy was related to IFNγ sensing by tumor cells and loss of PTPN2 can increase efficacy of immunotherapy by elevating the activity of IFNγ signaling (184). Furthermore, this group found that antagonist adenosine deaminase acting on RNA 1 (ADAR1) limited the sensing of endogenous double-stranded RNA (dsRNA), as an immune-suppressing molecule in the immune therapy of melanoma. Thus, loss of ADAR1 increased the sensing of IFN-inducible dsRNA, which enhanced tumor inflammation and strengthened the IFN sensitivity through MDA5 and PKR. Not surprisingly, disruption of ADAR1 overcomes resistance to PD-1 blockade caused by inactivation of antigen presentation by tumor cells (185). These studies demonstrate that IFNγ signaling plays important roles in resistance to ICB and targeting IFNγ signaling may improve clinical efficacies of immune checkpoint inhibitors.

This notion was further supported by the findings that loss of Janus kinase 1 (JAK1), a protein tyrosine kinase in the IFNγ pathway, induced resistance to T cell immune response elicited by blocking the PD-1 receptor (186). Patel et al. also identified another important regulator of resistance to checkpoint blockade, *i.e.* apelin receptor (*APLNR*), which interacts with JAK1 to modulate IFNγ responses in tumor cells (186). Its functional loss reduces the efficacy of ICB immunotherapies in mouse models. They also found multiple loss-of-function mutations in *APLNR* gene in patient tumor samples that might endow refractoriness to immunotherapy (204). In conclusion, the IFNγ signaling is emerging as a key player in resistance to checkpoint blockade therapy.

Besides, the core fucosylation pathway has been identified as positive regulator of cell-surface PD-1 expression through CRISPR-Cas9 screening (187). Similarly, CKLF like MARVEL transmembrane domain containing 6 (CMTM6) was identified as a regulator of cell surface PD-L1 expression by preventing its lysosomal degradation (186, 205). CMTM6 depletion significantly reactivated tumor-specific T cells *via* reducing PD-L1, indicating that CMTM6 could be a new drug target to enhance efficacy of checkpoint blockade (186, 205). In ALK positive anaplastic large-cell lymphoma, Zhang et al. found that interferon regulatory factor 4 (IRF4) and basic leucine zipper ATF-like transcription factor (BATF) increased PD-L1 expression by binding to the enhancer region of *PD-L1* gene. In contrast, growth factor receptor bound protein 2 (GRB2) and SOS Ras/Rac guanine nucleotide exchange factor 1 (SOS1) signalosome contribute to PD-L1 expression by activating the RAS-ERK and AKT pathways (206). Other study also found by CRISPR-Cas9 screening that targeting EGFR sensitized cancer cells to T-cell cytotoxicity. Thus, combination of PD-1 blockage with EGFR inhibition showed significant synergistic efficacy in a syngeneic model, further validating EGFR inhibitors as immunomodulatory agents for checkpoint blockade (207). Consistent with this notion, anti-angiogenesis therapy has been shown to enhance clinical efficacies of immune checkpoint blockade, which represent a combinatory therapeutic strategy to expand the landscape of cancer immunotherapy (208). In addition, Li et al. identified in an epigenetics-focused CRISPR-Cas9 screening that histone chaperone anti-silencing function 1A histone chaperone (ASF1A) was an important regulator of tumor immunity. Loss of ASF1A sensitized tumor cells to anti-PD-1 treatment by promoting M1-like macrophage polarization and T-cell activation (188). In conclusion, these previously unrecognized regulators not only accelerate our understanding of the molecular circuitry that drives tumor immune escape but also provide novel opportunities to improve immune checkpoint blockade strategies.

#### 4.1.6 The Mechanisms of Resistance to Tumor Vaccine and CAR-T Cell Therapies

Like other cancer treatments, cancer cells commonly develop resistance to tumor vaccine *via* different mechanisms. For instance, the increased number of regulatory T cells in cancer patients may constitute a resistance mechanism to the efficacy of cancer vaccine (209). Furthermore, Kimura et al. reported that high levels of myeloid-derived suppressive cells in patients correlated with resistance to immune response induced by cancer vaccine (210). These studies indicate that the induction of immunosuppressive environment in patients might cause resistance to cancer vaccine. Although there is no doubt that CRISPR-Cas9 screening provides a powerful tool to elucidate the mechanism of resistance to cancer therapies, more work is still needed to uncover its potential in unraveling the potential mechanism of resistance to tumor vaccine.

Cancer cells also develop resistance to CAR-T cell therapy in the clinic *via* the downregulation or loss of the targeted antigen (211, 212). The mechanisms to increase levels of the target antigen on the surface of cancer cells have the potential to restore efficacy to CAR-T cell therapy. For example, B-cell maturation antigen (BCMA) targeted CAR-T have shown improved responses in patients with relapsed and refractory multiple myeloma (MM). However, resistance and relapse to BCMA-targeted therapies have emerged as significant challenges and present an unmet need. To unravel the underlying resistance mechanisms, Ramkumar et al. performed CRISPR-Cas9 screening in a MM cell line, and identified several novel mechanisms regulate cell surface expression of the BCMA (213). Knockdown of genes in the sialic acid biosynthesis pathway sensitized MM cells to CAR-T-BCMA cells. Similarly, inhibition of HDAC7 and the Sec61 complex upregulates BCMA expression (213). Loss of function CRSIPR screening also showed that ICAM-1 expression is important for BCMA CAR T-cell–mediated tumor cell lysis, whereas knockdown of genes belonging to the family of diacylglycerol kinases increased sensitivity to BCMA CAR T cells (213). Interestingly, several studies showed that CRISPR/Cas9-mediated genome editing could augment the efficacy of CAR-T cell therapy by regulating the genes involved in the resistance to CAR-T cell therapy (214–216). Therefore, the CRISPR-Cas9 technology not only contributes to identifying novel targets for overcoming resistance to CAR T cell therapy, but also tackles resistance by editing these resistance related targets.

## 5 The Combined Application of the CRISPR-Cas9 Screening and Patient-Derived Xenograft or Organoid Model to Decipher Resistance Mechanisms

The traditional tumor cell line model lacks heterogeneity and the tumor microenvironment, which does not accurately reflect the biological characteristics of the original tumor. Patient-derived xenograft (PDX) model is constructed by transplanting tumor tissue from patient to immunocompromised mice, which circumvent the aforementioned shortcomings by maintaining genetic and cellular heterogeneity of tumor tissues. Similarly, patient-derived organoid (PDO) model could also faithfully recapitulate the molecular and phenotypic characteristics of human cancers. PDO is a microscopic self-organizing, three-dimensional organ-like structure generated from patient-derived stem cells *in vitro*. Basically, PDX and PDO have the following advantages over traditional cell line models: 1) High fidelity in maintaining the patient-specific genetic and cellular characteristics, 2) Efficient collection from patient and the ability of xenotransplantation, 3) Reliable drug sensitivity test results of the corresponding patient (217). These new models together with CRISPR-Cas9 screening significantly improve elucidation of resistance to existed therapies.

Using a PDX model, Grunblatt et al. identified the deubiquitinase USP7 as a MYCN-associated synthetic vulnerability in small cell lung cancer (SCLC) by a genome-scale CRISPR-Cas9 screening. Pharmacological inhibition of USP7 re-sensitized chemo-resistant MYCN-overexpressing PDX models to chemotherapy *in vivo* (218). Similarly, dihydroorotate dehydrogenase was also identified as a therapeutic target in SCLC cells (219). In another study, decapping enzyme scavenger (DCPS) was found to be an essential gene for the AML cells, while DCPS inhibitor (RG3039) suppressed the growth of AML in PDX model (220). Furthermore, using PDX models, the PRC2-NSD2/3-mediated MYC regulatory pathway has been identified as a drug-induced antagonistic pleiotropy pathway that confers resistance to bromodomain and BCL-2 inhibitors in AML (221). In searching for new therapeutic strategies against rhabdomyosarcoma (RMS), Bharathy et al. revealed that combination of Entinostat, a potent and selective inhibitor of class I and IV histone deacetylase (HDAC), with Vincristine exhibited enhanced antitumor activity in PDX models. Mechanistically, CRISPR-Cas9 screening revealed that HDAC3 was the major HDAC mediating therapeutic effects of Entinostat, leading to cell-autonomous cytoreduction of embryonal RMS (222). In a PDX model of pancreatic ductal adenocarcinoma, Szlachta et al. identified that several essential genes like *CENPE* and *NUF2* cause the resistance to MEK inhibition *via* large-scale CRISPR-Cas9 screening (223). The inhibition of these genes synergistically increases cellular sensitivity to MEK inhibition by regulating mitotic cell cycle and kinetochore function (223).

In CRC, the combination of CRISPR-Cas9 screening with PDO model has become an efficacious approach to identify key regulators in tumorigenesis. Michels et al. unraveled that transforming growth factor-beta receptor type 2 was the most common tumor suppressor in CRC cells (224). On the basis of CRISPR-Cas9 screening, importin-11 (IPO11) was found to be crucial for transcriptional activity of β-catenin in APC mutant CRC cells. Consistently, inhibition of IPO11 efficiently suppressed proliferation of CRC PDOs (225). Altogether, combination of patient-derived models and CRISPR-Cas9 screening provide more effective approaches to decipher the mechanisms of resistance to targeted cancer therapy and identify novel targets for cancer therapy.

## 6 Discussions and Future Perspectives

Molecular targeted therapies are widely applied in clinical setting, but drug resistance largely limits their clinical benefits. Therefore, understanding of underlying resistance mechanisms of these targeted therapies is utter important. Recent advances in gene editing technologies and research tools provide deeper insights into the resistance mechanism. For instance, CRISPR-Cas9 screening shows that deletion of genes like *KEAP1* and *ARIH*2 confer resistance to anti-EGFR therapy, indicating that inhibition of *KEAP1* and *ARIH*2 will enhance the efficacy of anti-EGFR targeted therapy (178, 192). Furthermore, the establishment of database of Cancer Dependency Map (DepMap) database has provide ample resource about potential dependency genes in different human cancer cell lines (154). Thus, restoration of these genes may specifically re-sensitize tumor cells to the treatment. In addition, loss of function mutations of these genes in tumor cells could also as potential biomarkers in predicting their therapeutic efficacies. On the other hand, ablation of oncogenes such as *RNF146* and *CLPB* in cancer cells could suppress tumor cell growth, thus sensitizing the cells to molecular targeted therapies (165, 177). Thus, targeting these oncogenic regulators may inhibit tumor growth and have a synergistic effect with molecular targeted drugs.

More recently, the emergence of patients-derived models including PDX and PDO makes it possible to perform functional CRISPR-Cas9 screening in the heterogeneous tumor mass. Thus, the combination of CRISPR-Cas9 screening with PDX/PDO model has emerged as a prevalent study mode. These efforts have led to novel findings of the mechanisms underpinning resistance of individual patients, which will undoubtedly pave the way for developing patient-specific precision medicine to combat various types of cancers. With the development of next generation PDX and PDO models that could fully recapitulate cancer immune environment, it is foreseeable that the combination of CRISPR-Cas9 screening and PDX/PDO models will further foster our understanding on the nature of tumor immunoevasion. In addition, zebrafish and fruit flies *in vivo* research systems have been utilized as novel models in cancer research, as they possess unique advantages like low-cost, easy husbandry and short life cycle (226–228). Recent studies have utilized *in vivo* CRISPR-Cas9 screening to identify key regulatory genes of development and tissue growth in zebrafish and drosophila (229, 230), indicating that similar approaches may be applicable to identify novel anti-cancer drug targets using these models, although experimental evidence is still lacking in this field. On the other hand, these models also have some limitations including body temperature and immunological systems differences with human, which need to be solved in the future in order to better understand the nature of human cancer. Nevertheless, these novel research models in combination with CRISPR-Cas9 screening will accelerate our understanding of drug resistance and identification of novel targets to improve cancer treatment.

## Author Contributions

JH and ZH wrote the manuscript. TL and DC provided technical support. BW, QW, and XZ conceived and supervised the study. All authors contributed to the article and approved the submitted version.

## Funding

This work was sponsored by the grants from the National Natural Science Foundation of China (NSFC Nos. 81822032, 91959111, and 81872027 to BW), Natural Science Foundation of Chongqing (No.cstc2021jcyj-msxmX0405 to XZ, No. CSTC2019JCYJJQX0027 to BW), fundings from Health Commission of Sichuan Province (17PJ575 to QLW) and Office of Science and Technology and Talent Work of Luzhou (2017LZXNYD-Z01 to QLW), and funding from the Army Medical University (Nos. 2019CXLCA001, 2018XLC2023 and 2019XQY19 to BW).

## Conflict of Interest

The authors declare that the research was conducted in the absence of any commercial or financial relationships that could be construed as a potential conflict of interest.

## Publisher’s Note

All claims expressed in this article are solely those of the authors and do not necessarily represent those of their affiliated organizations, or those of the publisher, the editors and the reviewers. Any product that may be evaluated in this article, or claim that may be made by its manufacturer, is not guaranteed or endorsed by the publisher.

## References

[1] Greenman C Stephens P Smith R Dalgliesh GL Hunter C Bignell G. Patterns of Somatic Mutation in Human Cancer Genomes. Nature (2007) 446(7132):153–8. doi: 10.1038/nature05610 PMC2712719 17344846

[2] Hanahan D Weinberg RJC. Hallmarks of Cancer: The Next Generation. Cell (2011) 144(5):646–74. doi: 10.1016/j.cell.2011.02.013 21376230

[3] Kim KJ Li B Winer J Armanini M Gillett N Phillips HS. Inhibition of Vascular Endothelial Growth Factor-Induced Angiogenesis Suppresses Tumour Growth In Vivo. Nature (1993) 362(6423):841–4. doi: 10.1038/362841a0 7683111

[4] Folkman J. Tumor Angiogenesis: Therapeutic Implications. N Engl J Med (1971) 285(21):1182–6. doi: 10.1056/NEJM197111182852108 4938153

[5] Folkman J Klagsbrun M. Angiogenic Factors. Science (1987) 235(4787):442–7. doi: 10.1126/science.2432664 2432664

[6] Wilke H Muro K Van Cutsem E Oh SC Bodoky G Shimada Y. Ramucirumab Plus Paclitaxel Versus Placebo Plus Paclitaxel in Patients With Previously Treated Advanced Gastric or Gastro-Oesophageal Junction Adenocarcinoma (RAINBOW): A Double-Blind, Randomised Phase 3 Trial. Lancet Oncol (2014) 15(11):1224–35. doi: 10.1016/S1470-2045(14)70420-6 25240821

[7] Van Cutsem E Tabernero J Lakomy R Prenen H Prausová J Macarulla T. Addition of Aflibercept to Fluorouracil, Leucovorin, and Irinotecan Improves Survival in a Phase III Randomized Trial in Patients With Metastatic Colorectal Cancer Previously Treated With an Oxaliplatin-Based Regimen. J Clin Oncol (2012) 30(28):3499–506. doi: 10.1200/JCO.2012.42.8201 22949147

[8] Robert C Long GV Brady B Dutriaux C Maio M Mortier L. Nivolumab in Previously Untreated Melanoma Without BRAF Mutation. N Engl J Med (2015) 372(4):320–30. doi: 10.1056/NEJMoa1412082 25399552

[9] Paz-Ares L Bálint B de Boer RH van Meerbeeck JP Wierzbicki R De Souza P. A Randomized Phase 2 Study of Paclitaxel and Carboplatin With or Without Conatumumab for First-Line Treatment of Advanced Non-Small-Cell Lung Cancer. J Thorac Oncol (2013) 8(3):329–37. doi: 10.1097/JTO.0b013e31827ce554 23370314

[10] Ahmed N Brawley VS Hegde M Robertson C Ghazi A Gerken C. Human Epidermal Growth Factor Receptor 2 (HER2) -Specific Chimeric Antigen Receptor-Modified T Cells for the Immunotherapy of HER2-Positive Sarcoma. J Clin Oncol (2015) 33(15):1688–96. doi: 10.1200/JCO.2014.58.0225 PMC4429176 25800760

[11] Baba Y Nosho K Shima K Meyerhardt JA Chan AT Engelman JA. Prognostic Significance of AMP-Activated Protein Kinase Expression and Modifying Effect of MAPK3/1 in Colorectal Cancer. Br J Cancer (2010) 103(7):1025–33. doi: 10.1038/sj.bjc.6605846 PMC2965861 20808308

[12] Marusyk A Almendro V Polyak K. Intra-Tumour Heterogeneity: A Looking Glass for Cancer? Nat Rev Cancer (2012) 12(5):323–34. doi: 10.1038/nrc3261 22513401

[13] Ramón YCS Sesé M Capdevila C Aasen T De Mattos-Arruda L Diaz-Cano SJ. Clinical Implications of Intratumor Heterogeneity: Challenges and Opportunities. J Mol Med (Berl) (2020) 98(2):161–77. doi: 10.1007/s00109-020-01874-2 PMC7007907 31970428

[14] Xu G McLeod HL. Strategies for Enzyme/Prodrug Cancer Therapy. Clin Cancer Res (2001) 7(11):3314–24. 11705842

[15] Padma VV. An Overview of Targeted Cancer Therapy. Biomed (Taipei) (2015) 5(4):19. doi: 10.7603/s40681-015-0019-4 PMC4662664 26613930

[16] Wong RS. Apoptosis in Cancer: From Pathogenesis to Treatment. J Exp Clin Cancer Res (2011) 30(1):87. doi: 10.1186/1756-9966-30-87 21943236 PMC3197541

[17] Tsujimoto Y Finger LR Yunis J Nowell PC Croce CM. Cloning of the Chromosome Breakpoint of Neoplastic B Cells With the T(14;18) Chromosome Translocation. Science (1984) 226(4678):1097–9. doi: 10.1126/science 6093263

[18] Stilgenbauer S Eichhorst B Schetelig J Hillmen P Seymour JF Coutre S. Venetoclax for Patients With Chronic Lymphocytic Leukemia With 17p Deletion: Results From the Full Population of a Phase II Pivotal Trial. J Clin Oncol (2018) 36(19):1973–80. doi: 10.1200/JCO 29715056

[19] Roberts AW Davids MS Pagel JM Kahl BS Puvvada SD Gerecitano JF. Targeting BCL2 With Venetoclax in Relapsed Chronic Lymphocytic Leukemia. N Engl J Med (2016) 374(4):311–22. doi: 10.1056/NEJMoa1513257 PMC7107002 26639348

[20] Casara P Davidson J Claperon A Le Toumelin-Braizat G Vogler M Bruno A. S55746 is a Novel Orally Active BCL-2 Selective and Potent Inhibitor That Impairs Hematological Tumor Growth. Oncotarget (2018) 9(28):20075–88. doi: 10.18632/oncotarget PMC5929447 29732004

[21] DiNardo CD Pratz K Pullarkat V Jonas BA Arellano M Becker PS. Venetoclax Combined With Decitabine or Azacitidine in Treatment-Naive, Elderly Patients With Acute Myeloid Leukemia. Blood (2019) 133(1):7–17. doi: 10.1182/blood-2018-08-868752 30361262 PMC6318429

[22] Oltersdorf T Elmore SW Shoemaker AR Armstrong RC Augeri DJ Belli BA. An Inhibitor of Bcl-2 Family Proteins Induces Regression of Solid Tumours. Nature (2005) 435(7042):677–81. doi: 10.1038/nature03579 15902208

[23] Roberts AW Seymour JF Brown JR Wierda WG Kipps TJ Khaw SL. Substantial Susceptibility of Chronic Lymphocytic Leukemia to BCL2 Inhibition: Results of a Phase I Study of Navitoclax in Patients With Relapsed or Refractory Disease. J Clin Oncol (2012) 30(5):488–96. doi: 10.1200/JCO.2011.34.7898 PMC4979082 22184378

[24] Kipps TJ Eradat H Grosicki S Catalano J Cosolo W Dyagil IS. A Phase 2 Study of the BH3 Mimetic BCL2 Inhibitor Navitoclax (ABT-263) With or Without Rituximab, in Previously Untreated B-Cell Chronic Lymphocytic Leukemia. Leuk Lymphoma (2015) 56(10):2826–33. doi: 10.3109/10428194.2015.1030638 PMC4643417 25797560

[25] Wertz IE Kusam S Lam C Okamoto T Sandoval W Anderson DJ. Sensitivity to Antitubulin Chemotherapeutics is Regulated by MCL1 and FBW7. Nature (2011) 471(7336):110–4. doi: 10.1038/nature09779 21368834

[26] Caenepeel S Brown SP Belmontes B Moody G Keegan KS Chui D. AMG 176, a Selective MCL1 Inhibitor, Is Effective in Hematologic Cancer Models Alone and in Combination With Established Therapies. Cancer Discovery (2018) 8(12):1582–97. 10.1158/2159-8290.CD-18-0387 30254093

[27] Tron AE Belmonte MA Adam A Aquila BM Boise LH Chiarparin E. Discovery of Mcl-1-Specific Inhibitor AZD5991 and Preclinical Activity in Multiple Myeloma and Acute Myeloid Leukemia. Nat Commun (2018) 9(1):5341. doi: 10.1038/s41467-018-07551-w 30559424 PMC6297231

[28] Itoh N Yonehara S Ishii A Yonehara M Mizushima S Sameshima M. The Polypeptide Encoded by the cDNA for Human Cell Surface Antigen Fas can Mediate Apoptosis. Cell (1991) 66(2):233–43. doi: 10.1016/0092-8674(91)90614-5 1713127

[29] Pennica D Nedwin GE Hayflick JS Seeburg PH Derynck R Palladino MA. Human Tumour Necrosis Factor: Precursor Structure, Expression and Homology to Lymphotoxin. Nature (1984) 312(5996):724–9. doi: 10.1038/312724a0 6392892

[30] Wakelee HA Patnaik A Sikic BI Mita M Fox NL Miceli R. Phase I and Pharmacokinetic Study of Lexatumumab (HGS-ETR2) Given Every 2 Weeks in Patients With Advanced Solid Tumors. Ann Oncol (2010) 21(2):376–81. doi: 10.1093/annonc/mdp292 PMC2813303 19633048

[31] Stein MN Malhotra J Tarapore RS Malhotra U Silk AW Chan N. Safety and Enhanced Immunostimulatory Activity of the DRD2 Antagonist ONC201 in Advanced Solid Tumor Patients With Weekly Oral Administration. J Immunother Cancer (2019) 7(1):136. doi: 10.1186/s40425-019-0599-8 31118108 PMC6532211

[32] Tahir SK Smith ML Solomon LR Zhang H Xue JC Xiao Y. Abbv-621 Is a Novel and Potent TRAIL Receptor Agonist Fusion Protein That Induces Apoptosis Alone and in Combination With Navitoclax and Venetoclax in Hematological Tumors. Blood (2017) 130(Supplement 1):2812–2. doi: 10.1182/blood.V130.Suppl_1.2812.2812

[33] Lim B Greer Y Lipkowitz S Takebe N. Novel Apoptosis-Inducing Agents for the Treatment of Cancer, a New Arsenal in the Toolbox. Cancers (Basel) (2019) 11(8):1087. doi: 10.3390/cancers11081087 PMC6721450 31370269

[34] Falschlehner C Ganten TM Koschny R Schaefer U Walczak H. TRAIL and Other TRAIL Receptor Agonists as Novel Cancer Therapeutics. Adv Exp Med Biol (2009) 647:195–206. doi: 10.1007/978-0-387-89520-8_14 19760076

[35] Plummer R Attard G Pacey S Li L Razak A Perrett R. Phase 1 and Pharmacokinetic Study of Lexatumumab in Patients With Advanced Cancers. Clin Cancer Res (2007) 13(20):6187–94. doi: 10.1158/1078-0432.CCR-07-0950 17947486

[36] Merchant MS Geller JI Baird K Chou AJ Galli S Charles A. Phase I Trial and Pharmacokinetic Study of Lexatumumab in Pediatric Patients With Solid Tumors. J Clin Oncol (2012) 30(33):4141–7. doi: 10.1200/JCO.2012.44.1055 PMC3494837 23071222

[37] Yue J López JM. Understanding MAPK Signaling Pathways in Apoptosis. Int J Mol Sci (2020) 21(7):2346. doi: 10.3390/ijms21072346 PMC7177758 32231094

[38] Sun Y Liu WZ Liu T Feng X Yang N Zhou HF. Signaling Pathway of MAPK/ERK in Cell Proliferation, Differentiation, Migration, Senescence and Apoptosis. J Recept Signal Transduct Res (2015) 35(6):600–4. doi: 10.3109/10799893.2015.1030412 26096166

[39] Santarpia L Lippman SM El-Naggar AK. Targeting the MAPK-RAS-RAF Signaling Pathway in Cancer Therapy. Expert Opin Ther Targets (2012) 16(1):103–19. doi: 10.1517/14728222.2011.645805 PMC3457779 22239440

[40] Kim KB Cabanillas ME Lazar AJ Williams MD Sanders DL Ilagan JL. Clinical Responses to Vemurafenib in Patients With Metastatic Papillary Thyroid Cancer Harboring BRAF(V600E) Mutation. Thyroid (2013) 23(10):1277–83. doi: 10.1089/thy.2013.0057 PMC3967415 23489023

[41] Kopetz S Desai J Chan E Hecht JR O'Dwyer PJ Lee RJ. PLX4032 in Metastatic Colorectal Cancer Patients With Mutant BRAF Tumors. (2010) 28(15_suppl):3534–4.

[42] Cheng Y Zhang G Li G. Targeting MAPK Pathway in Melanoma Therapy. Cancer Metastasis Rev (2013) 32(3-4):567–84. doi: 10.1007/s10555-013-9433-9 23584575

[43] Hertzman Johansson C Egyhazi Brage S. BRAF Inhibitors in Cancer Therapy. Pharmacol Ther (2014) 142(2):176–82. doi: 10.1016/j 24325952

[44] Neuzillet C Tijeras-Raballand A de Mestier L Cros J Faivre S Raymond E. MEK in Cancer and Cancer Therapy. Pharmacol Ther (2014) 141(2):160–71. doi: 10.1016/j.pharmthera.2013.10.001 24121058

[45] Sullivan RJ Infante JR Janku F Wong DJL Sosman JA Keedy V. First-In-Class ERK1/2 Inhibitor Ulixertinib (BVD-523) in Patients With MAPK Mutant Advanced Solid Tumors: Results of a Phase I Dose-Escalation and Expansion Study. Cancer Discov (2018) 8(2):184–95. doi: 10.1158/2159-8290.CD-17-1119 29247021

[46] Canon J Rex K Saiki AY Mohr C Cooke K Bagal D. The Clinical KRAS(G12C) Inhibitor AMG 510 Drives Anti-Tumour Immunity. Nature (2019) 575(7781):217–23. doi: 10.1038/s41586-019-1694-1 31666701

[47] Shanle EK Xu W. Selectively Targeting Estrogen Receptors for Cancer Treatment. Adv Drug Deliv Rev (2010) 62(13):1265–76. doi: 10.1016/j.addr.2010.08.001 PMC2991615 20708050

[48] Ahmed A Ali S Sarkar FH. Advances in Androgen Receptor Targeted Therapy for Prostate Cancer. J Cell Physiol (2014) 229(3):271–6. doi: 10.1002/jcp.24456 PMC3838461 24037862

[49] Tai W Mahato R Cheng K. The Role of HER2 in Cancer Therapy and Targeted Drug Delivery. J Control Release (2010) 146(3):264–75. doi: 10.1016/j.jconrel.2010.04.009 PMC2918695 20385184

[50] De Meyts P Whittaker J. Structural Biology of Insulin and IGF1 Receptors: Implications for Drug Design. Nat Rev Drug Discovery (2002) 1(10):769–83. doi: 10.1038/nrd917 12360255

[51] Pottier C Fresnais M Gilon M Jérusalem G Longuespée R Sounni NE. Tyrosine Kinase Inhibitors in Cancer: Breakthrough and Challenges of Targeted Therapy. Cancers (Basel) (2020) 12(3):731. doi: 10.3390/cancers12030731 PMC7140093 32244867

[52] Bennasroune A Gardin A Aunis D Crémel G Hubert P. Tyrosine Kinase Receptors as Attractive Targets of Cancer Therapy. Crit Rev Oncol Hematol (2004) 50(1):23–38. doi: 10.1016/j.critrevonc.2003.08.004 15094157

[53] Hojjat-Farsangi M. Small-Molecule Inhibitors of the Receptor Tyrosine Kinases: Promising Tools for Targeted Cancer Therapies. Int J Mol Sci (2014) 15(8):13768–801. doi: 10.3390/ijms150813768 PMC4159824 25110867

[54] Vanhaesebroeck B Guillermet-Guibert J Graupera M Bilanges B. The Emerging Mechanisms of Isoform-Specific PI3K Signalling. Nat Rev Mol Cell Biol (2010) 11(5):329–41. doi: 10.1038/nrm2882 20379207

[55] Dienstmann R Rodon J Serra V Tabernero J. Picking the Point of Inhibition: A Comparative Review of PI3K/AKT/mTOR Pathway Inhibitors. Mol Cancer Ther (2014) 13(5):1021–31. doi: 10.1158/1535-7163.MCT-13-0639 24748656

[56] Xie J Wang X Proud CG. mTOR Inhibitors in Cancer Therapy. F1000Res (2016) 5. doi: 10.12688/f1000research.9207.1 PMC5007757 27635236

[57] Hua H Kong Q Zhang H Wang J Luo T Jiang Y. Targeting mTOR for Cancer Therapy. J Hematol Oncol (2019) 12(1):71. doi: 10.1186/s13045-019-0754-1 31277692 PMC6612215

[58] Alzahrani AS. PI3K/Akt/mTOR Inhibitors in Cancer: At the Bench and Bedside. Semin Cancer Biol (2019) 59:125–32. doi: 10.1016/j.semcancer.2019.07.009 31323288

[59] Li J Kim SG Blenis J. Rapamycin: One Drug, Many Effects. Cell Metab (2014) 19(3):373–9. doi: 10.1016/j.cmet.2014.01.001 PMC3972801 24508508

[60] Wang Y Kim NS Haince JF Kang HC David KK Andrabi SA. Poly(ADP-Ribose) (PAR) Binding to Apoptosis-Inducing Factor Is Critical for PAR Polymerase-1-Dependent Cell Death (Parthanatos). Sci Signal (2011) 4(167):ra20. doi: 10.1126/scisignal.2000902 21467298 PMC3086524

[61] Yu SW Poitras MF Coombs C Bowers WJ Federoff HJ Poirier GG. Mediation of Poly(ADP-Ribose) Polymerase-1-Dependent Cell Death by Apoptosis-Inducing Factor. Science (2002) 297(5579):259–63. doi: 10.1126/science.1072221 12114629

[62] Faraoni I Graziani G. Role of BRCA Mutations in Cancer Treatment With Poly(ADP-Ribose) Polymerase (PARP) Inhibitors. Cancers (Basel) (2018) 10(12):487. doi: 10.3390/cancers10120487 PMC6316750 30518089

[63] Bryant HE Schultz N Thomas HD Parker KM Flower D Lopez E. Specific Killing of BRCA2-Deficient Tumours With Inhibitors of Poly(ADP-Ribose) Polymerase. Nature (2005) 434(7035):913–7. doi: 10.1038/nature03443 15829966

[64] Farmer H McCabe N Lord CJ Tutt AN Johnson DA Richardson TB. Targeting the DNA Repair Defect in BRCA Mutant Cells as a Therapeutic Strategy. Nature (2005) 434(7035):917–21. doi: 10.1038/nature03445 15829967

[65] Ledermann JA Harter P Gourley C Friedlander M Vergote I Rustin G. Overall Survival in Patients With Platinum-Sensitive Recurrent Serous Ovarian Cancer Receiving Olaparib Maintenance Monotherapy: An Updated Analysis From a Randomised, Placebo-Controlled, Double-Blind, Phase 2 Trial. Lancet Oncol (2016) 17(11):1579–89. doi: 10.1016/S1470-2045(16)30376-X 27617661

[66] Robson M Im SA Senkus E Xu B Domchek SM Masuda N. Olaparib for Metastatic Breast Cancer in Patients With a Germline BRCA Mutation. N Engl J Med (2017) 377(6):523–33. doi: 10.1056/NEJMoa1706450 28578601

[67] Patel M Nowsheen S Maraboyina S Xia F. The Role of Poly(ADP-Ribose) Polymerase Inhibitors in the Treatment of Cancer and Methods to Overcome Resistance: A Review. Cell Biosci (2020) 10:35. doi: 10.1186/s13578-020-00390-7 32180937 PMC7065339

[68] Heinicke U Haydn T Kehr S Vogler M Fulda S. BCL-2 Selective Inhibitor ABT-199 Primes Rhabdomyosarcoma Cells to Histone Deacetylase Inhibitor-Induced Apoptosis. Oncogene (2018) 37(39):5325–39. doi: 10.1038/s41388-018-0212-5 29858601

[69] Ramakrishnan VG Miller KC Macon EP Kimlinger TK Haug J Kumar S. Histone Deacetylase Inhibition in Combination With MEK or BCL-2 Inhibition in Multiple Myeloma. Haematologica (2019) 104(10):2061–74. doi: 10.3324/haematol.2018.211110 PMC6886422 30846494

[70] Sun K Atoyan R Borek MA Dellarocca S Rhyasen G Fattaey A. The Combination of Venetoclax and CUDC-907 Exhibits Synergistic Activity in Venetoclax-Refractory DLBCL. Blood (2016) 128(22):4184–4. doi: 10.1182/blood.V128.22.4184.4184

[71] Hasan A Haque E Hameed R Maier PN Irfan S Kamil M. Hsp90 Inhibitor Gedunin Causes Apoptosis in A549 Lung Cancer Cells by Disrupting Hsp90:Beclin-1:Bcl-2 Interaction and Downregulating Autophagy. Life Sci (2020) 256:118000. doi: 10.1016/j.lfs.2020.118000 32585246

[72] Parry D Guzi T Shanahan F Davis N Prabhavalkar D Wiswell D. Dinaciclib (SCH 727965), a Novel and Potent Cyclin-Dependent Kinase Inhibitor. Mol Cancer Ther (2010) 9(8):2344–53. doi: 10.1158/1535-7163.MCT-10-0324 20663931

[73] Feldmann G Mishra A Bisht S Karikari C Garrido-Laguna I Rasheed Z. Cyclin-Dependent Kinase Inhibitor Dinaciclib (SCH727965) Inhibits Pancreatic Cancer Growth and Progression in Murine Xenograft Models. Cancer Biol Ther (2011) 12(7):598–609. doi: 10.4161/cbt.12.7.16475 21768779 PMC3218385

[74] Gorlick R Kolb EA Houghton PJ Morton CL Neale G Keir ST. Initial Testing (Stage 1) of the Cyclin Dependent Kinase Inhibitor SCH 727965 (Dinaciclib) by the Pediatric Preclinical Testing Program. Pediatr Blood Cancer (2012) 59(7):1266–74. doi: 10.1002/pbc.24073 PMC3349821 22315240

[75] Arguello F Alexander M Sterry JA Tudor G Smith EM Kalavar NT. Flavopiridol Induces Apoptosis of Normal Lymphoid Cells, Causes Immunosuppression, and has Potent Antitumor Activity In Vivo Against Human Leukemia and Lymphoma Xenografts. Blood (1998) 91(7):2482–90. 9516149

[76] Stephenson JJ Nemunaitis J Joy AA Martin JC Jou YM Zhang D. Randomized Phase 2 Study of the Cyclin-Dependent Kinase Inhibitor Dinaciclib (MK-7965) Versus Erlotinib in Patients With non-Small Cell Lung Cancer. Lung Cancer (2014) 83(2):219–23. doi: 10.1016/j.lungcan.2013.11.020 24388167

[77] Gojo I Sadowska M Walker A Feldman EJ Iyer SP Baer MR. Clinical and Laboratory Studies of the Novel Cyclin-Dependent Kinase Inhibitor Dinaciclib (SCH 727965) in Acute Leukemias. Cancer Chemother Pharmacol (2013) 72(4):897–908. doi: 10.1007/s00280-013-2249-z 23949430 PMC3784060

[78] Mita MM Joy AA Mita A Sankhala K Jou YM Zhang D. Randomized Phase II Trial of the Cyclin-Dependent Kinase Inhibitor Dinaciclib (MK-7965) Versus Capecitabine in Patients With Advanced Breast Cancer. Clin Breast Cancer (2014) 14(3):169–76. doi: 10.1016/j.clbc.2013.10.016 24393852

[79] Sherr CJ Beach D Shapiro GI. Targeting CDK4 and CDK6: From Discovery to Therapy. Cancer Discov (2016) 6(4):353–67. doi: 10.1158/2159-8290.CD-15-0894 PMC4821753 26658964

[80] Fry DW Harvey PJ Keller PR Elliott WL Meade M Trachet E. Specific Inhibition of Cyclin-Dependent Kinase 4/6 by PD 0332991 and Associated Antitumor Activity in Human Tumor Xenografts. Mol Cancer Ther (2004) 3(11):1427–38. doi: 10.1158/1535-7163.1427.3.11 15542782

[81] Dean JL Thangavel C McClendon AK Reed CA Knudsen ES. Therapeutic CDK4/6 Inhibition in Breast Cancer: Key Mechanisms of Response and Failure. Oncogene (2010) 29(28):4018–32. doi: 10.1038/onc.2010.154 20473330

[82] Saab R Bills JL Miceli AP Anderson CM Khoury JD Fry DW. Pharmacologic Inhibition of Cyclin-Dependent Kinase 4/6 Activity Arrests Proliferation in Myoblasts and Rhabdomyosarcoma-Derived Cells. Mol Cancer Ther (2006) 5(5):1299–308. doi: 10.1158/1535-7163.MCT-05-0383 16731763

[83] Otto T Sicinski P. Cell Cycle Proteins as Promising Targets in Cancer Therapy. Nat Rev Cancer (2017) 17(2):93–115. doi: 10.1038/nrc.2016.138 28127048 PMC5345933

[84] Schenk EL Koh BD Flatten KS Peterson KL Parry D Hess AD. Effects of Selective Checkpoint Kinase 1 Inhibition on Cytarabine Cytotoxicity in Acute Myelogenous Leukemia Cells In Vitro. Clin Cancer Res (2012) 18(19):5364–73. doi: 10.1158/1078-0432.CCR-12-0961 PMC3463653 22869869

[85] Guzi TJ Paruch K Dwyer MP Labroli M Shanahan F Davis N. Targeting the Replication Checkpoint Using SCH 900776, a Potent and Functionally Selective CHK1 Inhibitor Identified via High Content Screening. Mol Cancer Ther (2011) 10(4):591–602. doi: 10.1158/1535-7163.MCT-10-0928 21321066

[86] Daud AI Ashworth MT Strosberg J Goldman JW Mendelson D Springett G. Phase I Dose-Escalation Trial of Checkpoint Kinase 1 Inhibitor MK-8776 as Monotherapy and in Combination With Gemcitabine in Patients With Advanced Solid Tumors. J Clin Oncol (2015) 33(9):1060–6. doi: 10.1200/JCO.2014.57.5027 25605849

[87] Karp JE Thomas BM Greer JM Sorge C Gore SD Pratz KW. Phase I and Pharmacologic Trial of Cytosine Arabinoside With the Selective Checkpoint 1 Inhibitor Sch 900776 in Refractory Acute Leukemias. Clin Cancer Res (2012) 18(24):6723–31. doi: 10.1158/1078-0432.CCR-12-2442 PMC3596113 23092873

[88] Corn PG Tu S-M Zurita AJ Subudhi SK Araujo JC Kim J. A Multi-Institutional Randomized Phase II Study (NCT01505868) of Cabazitaxel (CAB) Plus or Minus Carboplatin (CARB) in Men With Metastatic Castration-Resistant Prostate Cancer (mCRPC). J Clin Oncol (2015) 33(15_suppl):5010. doi: 10.1200/jco.2015.33.15_suppl.5010

[89] Oza AM Weberpals JI Provencher DM Grischke E-M Hall M Uyar D. An International, Biomarker-Directed, Randomized, Phase II Trial of AZD1775 Plus Paclitaxel and Carboplatin (P/C) for the Treatment of Women With Platinum-Sensitive, TP53-Mutant Ovarian Cancer. J Clin Oncol (2015) 33(15_suppl):5506. doi: 10.1200/jco.2015.33.15_suppl.5506

[90] Lugano R Ramachandran M Dimberg A. Tumor Angiogenesis: Causes, Consequences, Challenges and Opportunities. Cell Mol Life Sci (2020) 77(9):1745–70. doi: 10.1007/s00018-019-03351-7 PMC7190605 31690961

[91] Folkman J Merler E Abernathy C Williams G. Isolation of a Tumor Factor Responsible for Angiogenesis. J Exp Med (1971) 133(2):275–88. doi: 10.1084/jem.133.2.275 PMC2138906 4332371

[92] Hurwitz H Fehrenbacher L Novotny W Cartwright T Hainsworth J Heim W. Bevacizumab Plus Irinotecan, Fluorouracil, and Leucovorin for Metastatic Colorectal Cancer. N Engl J Med (2004) 350(23):2335–42. doi: 10.1056/NEJMoa032691 15175435

[93] Sandler A Gray R Perry MC Brahmer J Schiller JH Dowlati A. Paclitaxel-Carboplatin Alone or With Bevacizumab for Non-Small-Cell Lung Cancer. N Engl J Med (2006) 355(24):2542–50. doi: 10.1056/NEJMoa061884 17167137

[94] Shojaei F. Anti-Angiogenesis Therapy in Cancer: Current Challenges and Future Perspectives. Cancer Lett (2012) 320(2):130–7. doi: 10.1016/j.canlet.2012.03.008 22425960

[95] Bareschino MA Schettino C Troiani T Martinelli E Morgillo F Ciardiello F. Erlotinib in Cancer Treatment. Ann Oncol (2007) 18 Suppl 6:vi35–41. doi: 10.1093/annonc/mdm222 17591829

[96] Seshacharyulu P Ponnusamy MP Haridas D Jain M Ganti AK Batra SK. Targeting the EGFR Signaling Pathway in Cancer Therapy. Expert Opin Ther Targets (2012) 16(1):15–31. doi: 10.1517/14728222.2011.648617 22239438 PMC3291787

[97] Bokemeyer C Van Cutsem E Rougier P Ciardiello F Heeger S Schlichting M. Addition of Cetuximab to Chemotherapy as First-Line Treatment for KRAS Wild-Type Metastatic Colorectal Cancer: Pooled Analysis of the CRYSTAL and OPUS Randomised Clinical Trials. Eur J Cancer (2012) 48(10):1466–75. doi: 10.1016/j.ejca.2012.02.057 22446022

[98] Piessevaux H Buyse M Schlichting M Van Cutsem E Bokemeyer C Heeger S. Use of Early Tumor Shrinkage to Predict Long-Term Outcome in Metastatic Colorectal Cancer Treated With Cetuximab. J Clin Oncol (2013) 31(30):3764–75. doi: 10.1200/JCO.2012.42.8532 24043732

[99] Yazdi MH Faramarzi MA Nikfar S Abdollahi M. A Comprehensive Review of Clinical Trials on EGFR Inhibitors Such as Cetuximab and Panitumumab as Monotherapy and in Combination for Treatment of Metastatic Colorectal Cancer. Avicenna J Med Biotechnol (2015) 7(4):134–44. PMC4629455 26605007

[100] Maxwell PH Dachs GU Gleadle JM Nicholls LG Harris AL Stratford IJ. Hypoxia-Inducible Factor-1 Modulates Gene Expression in Solid Tumors and Influences Both Angiogenesis and Tumor Growth. Proc Natl Acad Sci USA (1997) 94(15):8104–9. doi: 10.1073/pnas.94.15.8104 PMC21564 9223322

[101] Skuli N Liu L Runge A Wang T Yuan L Patel S. Endothelial Deletion of Hypoxia-Inducible Factor-2alpha (HIF-2alpha) Alters Vascular Function and Tumor Angiogenesis. Blood (2009) 114(2):469–77. doi: 10.1182/blood-2008-12-193581 PMC2714217 19439736

[102] Coates JT Skwarski M Higgins GS. Targeting Tumour Hypoxia: Shifting Focus From Oxygen Supply to Demand. Br J Radiol (2019) 92(1093):20170843. doi: 10.1259/bjr.20170843 29436847 PMC6435066

[103] Ashton TM Fokas E Kunz-Schughart LA Folkes LK Anbalagan S Huether M. The Anti-Malarial Atovaquone Increases Radiosensitivity by Alleviating Tumour Hypoxia. Nat Commun (2016) 7:12308. doi: 10.1038/ncomms12308 27453292 PMC4962491

[104] Pandey A Shao H Marks RM Polverini PJ Dixit VM. Role of B61, the Ligand for the Eck Receptor Tyrosine Kinase, in TNF-Alpha-Induced Angiogenesis. Science (1995) 268(5210):567–9. doi: 10.1126/science.7536959 7536959

[105] Dobrzanski P Hunter K Jones-Bolin S Chang H Robinson C Pritchard S. Antiangiogenic and Antitumor Efficacy of EphA2 Receptor Antagonist. Cancer Res (2004) 64(3):910–9. doi: 10.1158/0008-5472.CAN-3430-2 14871820

[106] Fallah A Sadeghinia A Kahroba H Samadi A Heidari HR Bradaran B. Therapeutic Targeting of Angiogenesis Molecular Pathways in Angiogenesis-Dependent Diseases. BioMed Pharmacother (2019) 110:775–85. doi: 10.1016/j.biopha.2018.12.022 30554116

[107] Ferrara N Kerbel RS. Angiogenesis as a Therapeutic Target. Nature (2005) 438(7070):967–74. doi: 10.1038/nature04483 16355214

[108] Buchbinder EI Desai A. CTLA-4 and PD-1 Pathways: Similarities, Differences, and Implications of Their Inhibition. Am J Clin Oncol (2016) 39(1):98–106. doi: 10.1097/COC.0000000000000239 26558876 PMC4892769

[109] Leach DR Krummel MF Allison JP. Enhancement of Antitumor Immunity by CTLA-4 Blockade. Science (1996) 271(5256):1734–6. doi: 10.1126/science.271.5256.1734 8596936

[110] Hirano F Kaneko K Tamura H Dong H Wang S Ichikawa M. Blockade of B7-H1 and PD-1 by Monoclonal Antibodies Potentiates Cancer Therapeutic Immunity. Cancer Res (2005) 65(3):1089–96. doi: 10.1158/0008-5472.1089.65.3 15705911

[111] Hodi FS O'Day SJ McDermott DF Weber RW Sosman JA Haanen JB. Improved Survival With Ipilimumab in Patients With Metastatic Melanoma. N Engl J Med (2010) 363(8):711–23. doi: 10.1158/0008-5472.1089.65.3 PMC3549297 20525992

[112] Brahmer J Reckamp KL Baas P Crinò L Eberhardt WE Poddubskaya E. Nivolumab Versus Docetaxel in Advanced Squamous-Cell Non-Small-Cell Lung Cancer. N Engl J Med (2015) 373(2):123–35. doi: 10.1056/NEJMoa1504627 PMC4681400 26028407

[113] McDermott DF Drake CG Sznol M Choueiri TK Powderly JD Smith DC. Survival, Durable Response, and Long-Term Safety in Patients With Previously Treated Advanced Renal Cell Carcinoma Receiving Nivolumab. J Clin Oncol (2015) 33(18):2013–20. doi: 10.1200/JCO.2014.58.1041 PMC4517051 25800770

[114] Rotte A. Combination of CTLA-4 and PD-1 Blockers for Treatment of Cancer. J Exp Clin Cancer Res (2019) 38(1):255. doi: 10.1186/s13046-019-1259-z 31196207 PMC6567914

[115] Disselhorst MJ Quispel-Janssen J Lalezari F Monkhorst K de Vries JF van der Noort V. Ipilimumab and Nivolumab in the Treatment of Recurrent Malignant Pleural Mesothelioma (INITIATE): Results of a Prospective, Single-Arm, Phase 2 Trial. Lancet Respir Med (2019) 7(3):260–70. doi: 10.1016/S2213-2600(18)30420-X 30660511

[116] Scherpereel A Mazieres J Greillier L Lantuejoul S Dô P Bylicki O. Nivolumab or Nivolumab Plus Ipilimumab in Patients With Relapsed Malignant Pleural Mesothelioma (IFCT-1501 MAPS2): A Multicentre, Open-Label, Randomised, non-Comparative, Phase 2 Trial. Lancet Oncol (2019) 20(2):239–53. doi: 10.1016/S1470-2045(18)30765-4 30660609

[117] D'Angelo SP Mahoney MR Van Tine BA Atkins J Milhem MM Jahagirdar BN. Nivolumab With or Without Ipilimumab Treatment for Metastatic Sarcoma (Alliance A091401): Two Open-Label, non-Comparative, Randomised, Phase 2 Trials. Lancet Oncol (2018) 19(3):416–26. doi: 10.1016/S1470-2045(18)30006-8 PMC6126546 29370992

[118] Overman MJ McDermott R Leach JL Lonardi S Lenz HJ Morse MA. Nivolumab in Patients With Metastatic DNA Mismatch Repair-Deficient or Microsatellite Instability-High Colorectal Cancer (CheckMate 142): An Open-Label, Multicentre, Phase 2 Study. Lancet Oncol (2017) 18(9):1182–91. doi: 10.1016/S1470-2045(17)30422-9 PMC6207072 28734759

[119] Overman MJ Lonardi S Wong KYM Lenz HJ Gelsomino F Aglietta M. Durable Clinical Benefit With Nivolumab Plus Ipilimumab in DNA Mismatch Repair-Deficient/Microsatellite Instability-High Metastatic Colorectal Cancer. J Clin Oncol (2018) 36(8):773–9. doi: 10.1200/JCO.2017.76.9901 29355075

[120] Rosenberg JE Hoffman-Censits J Powles T van der Heijden MS Balar AV Necchi A. Atezolizumab in Patients With Locally Advanced and Metastatic Urothelial Carcinoma Who Have Progressed Following Treatment With Platinum-Based Chemotherapy: A Single-Arm, Multicentre, Phase 2 Trial. Lancet (2016) 387(10031):1909–20. doi: 10.1016/S0140-6736(16)00561-4 PMC5480242 26952546

[121] Waldman AD Fritz JM Lenardo MJ. A Guide to Cancer Immunotherapy: From T Cell Basic Science to Clinical Practice. Nat Rev Immunol (2020) 20(11):651–68. doi: 10.1038/s41577-020-0306-5 PMC7238960 32433532

[122] Mulati K Hamanishi J Matsumura N Chamoto K Mise N Abiko K. VISTA Expressed in Tumour Cells Regulates T Cell Function. Br J Cancer (2019) 120(1):115–27. doi: 10.1038/s41416-018-0313-5 PMC6325144 30382166

[123] Brignone C Gutierrez M Mefti F Brain E Jarcau R Cvitkovic F. First-Line Chemoimmunotherapy in Metastatic Breast Carcinoma: Combination of Paclitaxel and IMP321 (LAG-3Ig) Enhances Immune Responses and Antitumor Activity. J Transl Med (2010) 8:71. doi: 10.1186/1479-5876-8-71 20653948 PMC2920252

[124] Andrews LP Marciscano AE Drake CG Vignali DA. LAG3 (CD223) as a Cancer Immunotherapy Target. Immunol Rev (2017) 276(1):80–96. doi: 10.1111/imr.12519 28258692 PMC5338468

[125] Huang X Zhang X Li E Zhang G Wang X Tang T. VISTA: An Immune Regulatory Protein Checking Tumor and Immune Cells in Cancer Immunotherapy. J Hematol Oncol (2020) 13(1):83. doi: 10.1186/s13045-020-00917-y 32600443 PMC7325042

[126] Chen YT Scanlan MJ Sahin U Türeci O Gure AO Tsang S. A Testicular Antigen Aberrantly Expressed in Human Cancers Detected by Autologous Antibody Screening. Proc Natl Acad Sci USA (1997) 94(5):1914–8. doi: 10.1073/pnas.94.5.1914 PMC20017 9050879

[127] Acres B Limacher JM. MUC1 as a Target Antigen for Cancer Immunotherapy. Expert Rev Vaccines (2005) 4(4):493–502. doi: 10.1586/14760584.4.4.493 16117706

[128] Rosenberg SA Sherry RM Morton KE Scharfman WJ Yang JC Topalian SL. Tumor Progression can Occur Despite the Induction of Very High Levels of Self/Tumor Antigen-Specific CD8+ T Cells in Patients With Melanoma. J Immunol (2005) 175(9):6169–76. doi: 10.4049/jimmunol.175.9.6169 16237114

[129] Buonaguro L. Developments in Cancer Vaccines for Hepatocellular Carcinoma. Cancer Immunol Immunother (2016) 65(1):93–9. doi: 10.1007/s00262-015-1728-y PMC11028447 26093657

[130] Mittendorf EA Lu B Melisko M Price Hiller J Bondarenko I Brunt AM. Efficacy and Safety Analysis of Nelipepimut-S Vaccine to Prevent Breast Cancer Recurrence: A Randomized, Multicenter, Phase III Clinical Trial. Clin Cancer Res (2019) 25(14):4248–54. doi: 10.1158/1078-0432.CCR-18-2867 31036542

[131] Hollingsworth RE Jansen K. Turning the Corner on Therapeutic Cancer Vaccines. NPJ Vaccines (2019) 4:7. doi: 10.1038/s41541-019-0103-y 30774998 PMC6368616

[132] Vigneron N. Human Tumor Antigens and Cancer Immunotherapy. BioMed Res Int (2015) 2015:948501. doi: 10.1155/2015/948501 26161423 PMC4487697

[133] Hilf N Kuttruff-Coqui S Frenzel K Bukur V Stevanović S Gouttefangeas C. Actively Personalized Vaccination Trial for Newly Diagnosed Glioblastoma. Nature (2019) 565(7738):240–5. doi: 10.1038/s41586-018-0810-y 30568303

[134] Strønen E Toebes M Kelderman S van Buuren Yang W van Rooij N. Targeting of Cancer Neoantigens With Donor-Derived T Cell Receptor Repertoires. Science (2016) 352(6291):1337–41. doi: 10.1126/science.aaf2288 27198675

[135] Sahin U Derhovanessian E Miller M Kloke BP Simon P Löwer M. Personalized RNA Mutanome Vaccines Mobilize Poly-Specific Therapeutic Immunity Against Cancer. Nature (2017) 547(7662):222–6. doi: 10.1038/nature23003 28678784

[136] Kochenderfer JN Wilson WH Janik JE Dudley ME Stetler-Stevenson M Feldman SA. Eradication of B-Lineage Cells and Regression of Lymphoma in a Patient Treated With Autologous T Cells Genetically Engineered to Recognize CD19. Blood (2010) 116(20):4099–102. doi: 10.1182/blood-2010-04-281931 PMC2993617 20668228

[137] Porter DL Levine BL Kalos M Bagg A June CH. Chimeric Antigen Receptor-Modified T Cells in Chronic Lymphoid Leukemia. N Engl J Med (2011) 365(8):725–33. doi: 10.1056/NEJMoa1103849 PMC3387277 21830940

[138] Shah NN Fry TJ. Mechanisms of Resistance to CAR T Cell Therapy. Nat Rev Clin Oncol (2019) 16(6):372–85. doi: 10.1038/s41571-019-0184-6 PMC8214555 30837712

[139] Maus MV. Designing CAR T Cells for Glioblastoma. Oncoimmunology (2015) 4(12):e1048956. 26587317 10.1080/2162402X.2015.1048956 PMC4635938

[140] Morgan RA Johnson LA Davis JL Zheng Z Woolard KD Reap EA. Recognition of Glioma Stem Cells by Genetically Modified T Cells Targeting EGFRvIII and Development of Adoptive Cell Therapy for Glioma. Hum Gene Ther (2012) 23(10):1043–53. doi: 10.1089/hum.2012.041 PMC3472555 22780919

[141] Ahmed N Ratnayake M Savoldo B Perlaky L Dotti G Wels WS. Regression of Experimental Medulloblastoma Following Transfer of HER2-Specific T Cells. Cancer Res (2007) 67(12):5957–64. doi: 10.1158/0008-5472.CAN-06-4309 17575166

[142] Ahmed N Salsman VS Yvon E Louis CU Perlaky L Wels WS. Immunotherapy for Osteosarcoma: Genetic Modification of T Cells Overcomes Low Levels of Tumor Antigen Expression. Mol Ther (2009) 17(10):1779–87. doi: 10.1038/mt.2009.133 PMC2835000 19532139

[143] Koneru M Purdon TJ Spriggs D Koneru S Brentjens RJ. IL-12 Secreting Tumor-Targeted Chimeric Antigen Receptor T Cells Eradicate Ovarian Tumors In Vivo. Oncoimmunology (2015) 4(3):e994446. doi: 10.4161/2162402X.2014.994446 25949921 PMC4404840

[144] Koneru M O'Cearbhaill R Pendharkar S Spriggs DR Brentjens RJ. A Phase I Clinical Trial of Adoptive T Cell Therapy Using IL-12 Secreting MUC-16(Ecto) Directed Chimeric Antigen Receptors for Recurrent Ovarian Cancer. J Transl Med (2015) 13:102. doi: 10.1186/s12967-015-0460-x 25890361 PMC4438636

[145] Chen C Li K Jiang H Song F Gao H Pan X. Development of T Cells Carrying Two Complementary Chimeric Antigen Receptors Against Glypican-3 and Asialoglycoprotein Receptor 1 for the Treatment of Hepatocellular Carcinoma. Cancer Immunol Immunother (2017) 66(4):475–89. doi: 10.1007/s00262-016-1949-8 PMC11028818 28035433

[146] Kahraman C Kahraman NK Aras B Coşgun S Gülcan E. The Relationship Between Neutrophil-to-Lymphocyte Ratio and Albuminuria in Type 2 Diabetic Patients: A Pilot Study. Arch Med Sci (2016) 12(3):571–5. doi: 10.5114/aoms.2016.59931 PMC4889692 27279850

[147] Lovly CM Shaw AT. Molecular Pathways: Resistance to Kinase Inhibitors and Implications for Therapeutic Strategies. Clin Cancer Res (2014) 20(9):2249–56. doi: 10.1158/1078-0432.CCR-13-1610 PMC4029617 24789032

[148] Benedettini E Sholl LM Peyton M Reilly J Ware C Davis L. Met Activation in non-Small Cell Lung Cancer is Associated With De Novo Resistance to EGFR Inhibitors and the Development of Brain Metastasis. Am J Pathol (2010) 177(1):415–23. doi: 10.2353/ajpath.2010.090863 PMC2893683 20489150

[149] Han SH Korm S Han YG Choi SY Kim SH Chung HJ. GCA Links TRAF6-ULK1-Dependent Autophagy Activation in Resistant Chronic Myeloid Leukemia. Autophagy (2019) 15(12):2076–90. doi: 10.1080/15548627.2019.1596492 PMC6844495 30929559

[150] Shalem O Sanjana NE Hartenian E Shi X Scott DA Mikkelson T. Genome-Scale CRISPR-Cas9 Knockout Screening in Human Cells. Science (2014) 343(6166):84–7. doi: 10.1126/science.1247005 PMC4089965 24336571

[151] Sharma S Petsalaki E. Application of CRISPR-Cas9 Based Genome-Wide Screening Approaches to Study Cellular Signalling Mechanisms. Int J Mol Sci (2018) 19(4):933. doi: 10.3390/ijms19040933 PMC5979383 29561791

[152] Hart T Chandrashekhar M Aregger M Steinhart Z Brown KR MacLeod G. High-Resolution CRISPR Screens Reveal Fitness Genes and Genotype-Specific Cancer Liabilities. Cell (2015) 163(6):1515–26. doi: 10.1016/j.cell.2015.11.015 26627737

[153] Wang T Birsoy K Hughes NW Krupczak KM Post Y Wei JJ. Identification and Characterization of Essential Genes in the Human Genome. Science (2015) 350(6264):1096–101. doi: 10.1126/science.aac7041 PMC4662922 26472758

[154] Tsherniak A Vazquez F Montgomery PG Weir BA Kryukov G Cowley GS. Defining a Cancer Dependency Map. Cell (2017) 170(3):564–76.e16. 28753430 10.1016/j.cell.2017.06.010 PMC5667678

[155] Liao S Davoli T Leng Y Li MZ Xu Q Elledge SJ. A Genetic Interaction Analysis Identifies Cancer Drivers That Modify EGFR Dependency. Genes Dev (2017) 31(2):184–96. doi: 10.1101/gad.291948.116 PMC5322732 28167502

[156] Georgiou A Stewart A Cunningham D Banerji U Whittaker SR. Inactivation of NF1 Promotes Resistance to EGFR Inhibition in KRAS/NRAS/BRAF(V600) -Wild-Type Colorectal Cancer. Mol Cancer Res (2020) 18(6):835–46. doi: 10.1158/1541-7786.MCR-19-1201 PMC7611272 32098826

[157] Chen J Bell J Lau BT Whittaker T Stapleton D Ji HP. A Functional CRISPR/Cas9 Screen Identifies Kinases That Modulate FGFR Inhibitor Response in Gastric Cancer. Oncogenesis (2019) 8(5):33. doi: 10.1038/s41389-019-0145-z 31076567 PMC6510732

[158] Dompe N Klijn C Watson SA Leng K Port J Cuellar T. A CRISPR Screen Identifies MAPK7 as a Target for Combination With MEK Inhibition in KRAS Mutant NSCLC. PloS One (2018) 13(6):e0199264. doi: 10.1371/journal.pone.0199264 29912950 PMC6005515

[159] Wang B Krall EB Aguirre AJ Kim M Widlund HR Doshi MB. ATXN1L, CIC, and ETS Transcription Factors Modulate Sensitivity to MAPK Pathway Inhibition. Cell Rep (2017) 18(6):1543–57. doi: 10.1016/j.celrep.2017.01.031 PMC5313047 28178529

[160] Nagler A Vredevoogd DW Alon M Cheng PF Trabish S Kalaora S. A Genome-Wide CRISPR Screen Identifies FBXO42 Involvement in Resistance Toward MEK Inhibition in NRAS-Mutant Melanoma. Pigment Cell Melanoma Res (2020) 33(2):334–44. doi: 10.1111/pcmr.12825 PMC7383499 31549767

[161] Yau EH Kummetha IR Lichinchi G Tang R Zhang Y Rana TM. Genome-Wide CRISPR Screen for Essential Cell Growth Mediators in Mutant KRAS Colorectal Cancers. Cancer Res (2017) 77(22):6330–9. doi: 10.1158/0008-5472.CAN-17-2043 PMC5690866 28954733

[162] Sharon D Cathelin S Mirali S Di Trani JM Yanofsky DJ Keon KA. Inhibition of Mitochondrial Translation Overcomes Venetoclax Resistance in AML Through Activation of the Integrated Stress Response. Sci Transl Med (2019) 11(516):eaax2863. doi: 10.1126/scitranslmed.aax2863 31666400

[163] Schettini F De Santo I Rea CG De Placido P Formisano L Giuliano M. CDK 4/6 Inhibitors as Single Agent in Advanced Solid Tumors. Front Oncol (2018) 8:608. doi: 10.3389/fonc.2018.00608 30631751 PMC6315195

[164] Martin TD Cook DR Choi MY Li MZ Haigis KM Elledge SJ. A Role for Mitochondrial Translation in Promotion of Viability in K-Ras Mutant Cells. Cell Rep (2017) 20(2):427–38. doi: 10.1016/j.celrep.2017.06.061 PMC5553568 28700943

[165] Chen X Glytsou C Zhou H Narang S Reyna DE Lopez A. Targeting Mitochondrial Structure Sensitizes Acute Myeloid Leukemia to Venetoclax Treatment. Cancer Discovery (2019) 9(7):890–909. doi: 10.1158/2159-8290 31048321 PMC6606342

[166] Dev H Chiang TW Lescale C de Krijger I Martin AG Pilger D. Shieldin Complex Promotes DNA End-Joining and Counters Homologous Recombination in BRCA1-Null Cells. Nat Cell Biol (2018) 20(8):954–65. doi: 10.1038/s41556-018-0140-1 PMC6145444 30022119

[167] Zhao Q Lan T Su S Rao Y. Induction of Apoptosis in MDA-MB-231 Breast Cancer Cells by a PARP1-Targeting PROTAC Small Molecule. Chem Commun (Camb) (2019) 55(3):369–72. doi: 10.1039/C8CC07813K 30540295

[168] Zimmermann M Murina O Reijns MAM Agathanggelou A Challis R Tarnauskaitė Ž. CRISPR Screens Identify Genomic Ribonucleotides as a Source of PARP-Trapping Lesions. Nature (2018) 559(7713):285–9. doi: 10.1038/s41586-018-0291-z PMC6071917 29973717

[169] Tang H De Matos Simoes R Shirasaki R Dashevsky O Glassner B Sondra L. CRISPR Activation Screen for HDAC Inhibitor Resistance. Blood (2018) 132(Supplement 1):3958–8. doi: 10.1182/blood-2018-99-119044

[170] Chou TC. Theoretical Basis, Experimental Design, and Computerized Simulation of Synergism and Antagonism in Drug Combination Studies. Pharmacol Rev (2006) 58(3):621–81. doi: 10.1124/pr.58.3.10 16968952

[171] Hayes TK Luo F Cohen O Goodale AB Lee Y Pantel S. A Functional Landscape of Resistance to MEK1/2 and CDK4/6 Inhibition in NRAS-Mutant Melanoma. Cancer Res (2019) 79(9):2352–66. doi: 10.1158/0008-5472.CAN-18-2711 PMC7227487 30819666

[172] Ding Y Gong C Huang D Chen R Sui P Lin KH. Synthetic Lethality Between HER2 and Transaldolase in Intrinsically Resistant HER2-Positive Breast Cancers. Nat Commun (2018) 9(1):4274. doi: 10.1038/s41467-018-06651-x 30323337 PMC6189078

[173] Strub T Ghiraldini FG Carcamo S Li M Wroblewska A Singh R. SIRT6 Haploinsufficiency Induces BRAF(V600E) Melanoma Cell Resistance to MAPK Inhibitors via IGF Signalling. Nat Commun (2018) 9(1):3440. doi: 10.1038/s41467-018-05966-z 30143629 PMC6109055

[174] Jayson GC Ghiraldini FG Carcamo S Li M Wroblewska A Singh R. Antiangiogenic Therapy in Oncology: Current Status and Future Directions. Lancet (2016) 388(10043):518–29. doi: 10.1016/S0140-6736(15)01088-0 26853587

[175] Zhao D Zhai B He C Tan G Jiang X Pan S. Upregulation of HIF-2α Induced by Sorafenib Contributes to the Resistance by Activating the TGF-α/EGFR Pathway in Hepatocellular Carcinoma Cells. Cell Signal (2014) 26(5):1030–9. doi: 10.1016/j.cellsig.2014.01.026 24486412

[176] Liang Y Zheng T Song R Wang J Yin D Wang L. Hypoxia-Mediated Sorafenib Resistance can be Overcome by EF24 Through Von Hippel-Lindau Tumor Suppressor-Dependent HIF-1α Inhibition in Hepatocellular Carcinoma. Hepatology (2013) 57(5):1847–57. doi: 10.1002/hep.26224 23299930

[177] Wang H Lu B Castillo J Zhang Y Yang Z McAllister G. Tankyrase Inhibitor Sensitizes Lung Cancer Cells to Endothelial Growth Factor Receptor (EGFR) Inhibition via Stabilizing Angiomotins and Inhibiting YAP Signaling. J Biol Chem (2016) 291(29):15256–66. doi: 10.1074/jbc.M116.722967 PMC4946938 27231341

[178] Zeng H Castillo-Cabrera J Manser M Lu B Yang Z Strande V. Genome-Wide CRISPR Screening Reveals Genetic Modifiers of Mutant EGFR Dependence in Human NSCLC. Elife (2019) 8. doi: 10.7554/eLife.50223 PMC6927754 31741433

[179] Bunda S Heir P Metcalf J Li ASC Agnihotri S Pusch S. CIC Protein Instability Contributes to Tumorigenesis in Glioblastoma. Nat Commun (2019) 10(1):661. doi: 10.1038/s41467-018-08087-9 30737375 PMC6368580

[180] Kiessling MK Schuierer S Stertz S Beibel M Bergling S Knehr J. Identification of Oncogenic Driver Mutations by Genome-Wide CRISPR-Cas9 Dropout Screening. BMC Genomics (2016) 17(1):723. doi: 10.1186/s12864-016-3042-2 27613601 PMC5016932

[181] Sun W He B Yang B Hu W Cheng S Xiao H. Genome-Wide CRISPR Screen Reveals SGOL1 as a Druggable Target of Sorafenib-Treated Hepatocellular Carcinoma. Lab Invest (2018) 98(6):734–44. doi: 10.1038/s41374-018-0027-6 29467456

[182] Wang C He B Yang B Hu W Cheng S Xiao H. CDK12 Inhibition Mediates DNA Damage and is Synergistic With Sorafenib Treatment in Hepatocellular Carcinoma. Gut (2020) 69(4):727–36. doi: 10.1136/gutjnl-2019-318506 31519701

[183] Zheng A Chevalier N Calderoni M Dubuis G Dormond O Ziros PG. CRISPR/Cas9 Genome-Wide Screening Identifies KEAP1 as a Sorafenib, Lenvatinib, and Regorafenib Sensitivity Gene in Hepatocellular Carcinoma. Oncotarget (2019) 10(66):7058–70. doi: 10.18632/oncotarget.27361 PMC6925031 31903165

[184] Manguso RT Pope HW Zimmer MD Brown FD Yates KB Miller BC. In Vivo CRISPR Screening Identifies Ptpn2 as a Cancer Immunotherapy Target. Nature (2017) 547(7664):413–8. doi: 10.1038/nature23270 PMC5924693 28723893

[185] Ishizuka JJ Manguso RT Cheruiyot CK Bi K Panda A Iracheta-Vellve A. Loss of ADAR1 in Tumours Overcomes Resistance to Immune Checkpoint Blockade. Nature (2019) 565(7737):43–8. doi: 10.1038/s41586-018-0768-9 PMC7241251 30559380

[186] Han P Dai Q Fan L Lin H Zhang X Li F. Genome-Wide CRISPR Screening Identifies JAK1 Deficiency as a Mechanism of T-Cell Resistance. Front Immunol (2019) 10:251. doi: 10.3389/fimmu.2019.00251 30837996 PMC6389627

[187] Okada M Chikuma S Kondo T Hibino S Machiyama H Yokosuka T. Blockage of Core Fucosylation Reduces Cell-Surface Expression of PD-1 and Promotes Anti-Tumor Immune Responses of T Cells. Cell Rep (2017) 20(5):1017–28. doi: 10.1016/j.celrep.2017.07.027 28768188

[188] Li F Huang Q Luster TA Hu H Zhang H Ng WL. In Vivo Epigenetic CRISPR Screen Identifies Asf1a as an Immunotherapeutic Target in Kras-Mutant Lung Adenocarcinoma. Cancer Discov (2020) 10(2):270–87. doi: 10.1158/2159-8290.CD-19-0780 PMC7007372 31744829

[189] Šuštić T van Wageningen S Bosdriesz E Reid RJD Dittmar J Lieftink C. A Role for the Unfolded Protein Response Stress Sensor ERN1 in Regulating the Response to MEK Inhibitors in KRAS Mutant Colon Cancers. Genome Med (2018) 10(1):90. doi: 10.1186/s13073-018-0600-z 30482246 PMC6258447

[190] Sulahian R Kwon JJ Walsh KH Pailler E Bosse TL Thaker M. Synthetic Lethal Interaction of SHOC2 Depletion With MEK Inhibition in RAS-Driven Cancers. Cell Rep (2019) 29(1):118–34.e8. doi: 10.1016/j.celrep.2019.08.090 31577942 PMC6918830

[191] Milton CK Self AJ Clarke PA Banerji U Piccioni F Root DE. A Genome-Scale CRISPR Screen Identifies the ERBB and mTOR Signaling Networks as Key Determinants of Response to PI3K Inhibition in Pancreatic Cancer. Mol Cancer Ther (2020) 19(7):1423–35. 10.1158/1535-7163.MCT-19-1131 PMC7611271 32371585

[192] Krall EB Wang B Munoz DM Ilic N Raghavan S Niederst MJ. Correction: KEAP1 Loss Modulates Sensitivity to Kinase Targeted Therapy in Lung Cancer. Elife (2017) 6. 10.7554/eLife.18970 PMC5305212 28145866

[193] Fang P De Souza C Minn K Chien J. Genome-Scale CRISPR Knockout Screen Identifies TIGAR as a Modifier of PARP Inhibitor Sensitivity. Commun Biol (2019) 2:335. doi: 10.1038/s42003-019-0580-6 31508509 PMC6733792

[194] Finn RS Martin M Rugo HS Jones S Im SA Gelmon K. Palbociclib and Letrozole in Advanced Breast Cancer. N Engl J Med (2016) 375(20):1925–36. doi: 10.1056/NEJMoa1607303 27959613

[195] Hortobagyi GN Stemmer SM Burris HA Yap YS Sonke GS Paluch-Shimon S. Ribociclib as First-Line Therapy for HR-Positive, Advanced Breast Cancer. N Engl J Med (2016) 375(18):1738–48. doi: 10.1056/NEJMoa1609709 27717303

[196] Tong Z Sathe A Ebner B Qi P Veltkamp C Gschwend JE. Functional Genomics Identifies Predictive Markers and Clinically Actionable Resistance Mechanisms to CDK4/6 Inhibition in Bladder Cancer. J Exp Clin Cancer Res (2019) 38(1):322. doi: 10.1186/s13046-019-1322-9 31331377 PMC6647307

[197] Terai H Kitajima S Potter DS Matsui Y Quiceno LG Chen T. ER Stress Signaling Promotes the Survival of Cancer "Persister Cells" Tolerant to EGFR Tyrosine Kinase Inhibitors. Cancer Res (2018) 78(4):1044–57. doi: 10.1158/0008-5472.CAN-17-1904 PMC5815936 29259014

[198] Hay DA Fedorov O Martin S Singleton DC Tallant C Wells C. Discovery and Optimization of Small-Molecule Ligands for the CBP/p300 Bromodomains. J Am Chem Soc (2014) 136(26):9308–19. doi: 10.1021/ja412434f PMC4183655 24946055

[199] Robey RW Chakraborty AR Basseville A Luchenko V Bahr J Zhan Z. Histone Deacetylase Inhibitors: Emerging Mechanisms of Resistance. Mol Pharm (2011) 8(6):2021–31. doi: 10.1021/mp200329f PMC3230675 21899343

[200] Fantin VR Richon VM. Mechanisms of Resistance to Histone Deacetylase Inhibitors and Their Therapeutic Implications. Clin Cancer Res (2007) 13(24):7237–42. 10.1158/1078-0432.CCR-07-2114 18094401

[201] Wei L Lee D Law CT Zhang MS Shen J Chin DW. Genome-Wide CRISPR/Cas9 Library Screening Identified PHGDH as a Critical Driver for Sorafenib Resistance in HCC. Nat Commun (2019) 10(1):4681. doi: 10.1038/s41467-019-12606-7 31615983 PMC6794322

[202] Suemura S Kodama T Myojin Y Yamada R Shigekawa M Hikita H. CRISPR Loss-Of-Function Screen Identifies the Hippo Signaling Pathway as the Mediator of Regorafenib Efficacy in Hepatocellular Carcinoma. Cancers (Basel) (2019) 11(9):1362. doi: 10.3390/cancers11091362. PMC6770429 31540262

[203] Gao J Shi LZ Zhao H Chen J Xiong L He Q. Loss of IFN-γ Pathway Genes in Tumor Cells as a Mechanism of Resistance to Anti-CTLA-4 Therapy. Cell (2016) 167(2):397–404.e9. doi: 10.1016/j.cell.2016.08.069 27667683 PMC5088716

[204] Patel SJ Sanjana NE Kishton RJ Eidizadeh A Vodnala SK Cam M. Identification of Essential Genes for Cancer Immunotherapy. Nature (2017) 548(7669):537–42. doi: 10.1038/nature23477 PMC5870757 28783722

[205] Burr ML Sparbier CE Chan YC Williamson JC Woods K Beavis PA. CMTM6 Maintains the Expression of PD-L1 and Regulates Anti-Tumour Immunity. Nature (2017) 549(7670):101–5. doi: 10.1038/nature23643 PMC5706633 28813417

[206] Zhang JP Song Z Wang HB Lang L Yang YZ Xiao W. A Novel Model of Controlling PD-L1 Expression in ALK(+) Anaplastic Large Cell Lymphoma Revealed by CRISPR Screening. Blood (2019) 134(2):171–85. doi: 10.1182/blood.2019001043 PMC6624970 31151983

[207] Lizotte PH Hong RL Luster TA Cavanaugh ME Taus LJ Wang S. A High-Throughput Immune-Oncology Screen Identifies EGFR Inhibitors as Potent Enhancers of Antigen-Specific Cytotoxic T-Lymphocyte Tumor Cell Killing. Cancer Immunol Res (2018) 6(12):1511–23. doi: 10.1158/2326-6066 PMC6601346 30242021

[208] Lee WS Yang H Chon HJ Kim C. Combination of Anti-Angiogenic Therapy and Immune Checkpoint Blockade Normalizes Vascular-Immune Crosstalk to Potentiate Cancer Immunity. Exp Mol Med (2020) 52(9):1475–85. doi: 10.1038/s12276-020-00500-y PMC8080646 32913278

[209] Pere H Tanchot C Bayry J Terme M Taieb J Badoual C. Comprehensive Analysis of Current Approaches to Inhibit Regulatory T Cells in Cancer. Oncoimmunology (2012) 1(3):326–33. doi: 10.4161/onci.18852 PMC3382865 22737608

[210] Kimura T McKolanis JR Dzubinski LA Islam K Potter DM Salazar AM. MUC1 Vaccine for Individuals With Advanced Adenoma of the Colon: A Cancer Immunoprevention Feasibility Study. Cancer Prev Res (Phila) (2013) 6(1):18–26. doi: 10.1158/1940-6207.CAPR-12-0275 23248097 PMC3536916

[211] Majzner RG Mackall CL. Tumor Antigen Escape From CAR T-Cell Therapy. Cancer Discovery (2018) 8(10):1219–26. doi: 10.1158/2159-8290.CD-18-0442 30135176

[212] Iorgulescu JB Braun D Oliveira G Keskin DB Wu CJ. Acquired Mechanisms of Immune Escape in Cancer Following Immunotherapy. Genome Med (2018) 10(1):87. doi: 10.1186/s13073-018-0598-2 30466478 PMC6249768

[213] Ramkumar P Abarientos AB Tian R Seyler M Leong JT Chen M. CRISPR-Based Screens Uncover Determinants of Immunotherapy Response in Multiple Myeloma - ScienceDirect. Blood Adv (2020) 4(13):2899–911. doi: 10.1101/833707 PMC7362346 32589729

[214] Nakazawa T Natsume A Nishimura F Morimoto T Matsuda R Nakamura M. Effect of CRISPR/Cas9-Mediated PD-1-Disrupted Primary Human Third-Generation CAR-T Cells Targeting EGFRvIII on In Vitro Human Glioblastoma Cell Growth. Cells (2020) 9(4):998. doi: 10.3390/cells9040998 PMC7227242 32316275

[215] Tang N Cheng C Zhang X Qiao M Li N Mu W. TGF-β Inhibition via CRISPR Promotes the Long-Term Efficacy of CAR T Cells Against Solid Tumors. JCI Insight (2020) 5(4):e133977. doi: 10.1172/jci.insight.133977 PMC7101140 31999649

[216] Jung IY Kim YY Yu HS Lee M Kim S Lee J. CRISPR/Cas9-Mediated Knockout of DGK Improves Antitumor Activities of Human T Cells. Cancer Res (2018) 78(16):4692–703. doi: 10.1158/0008-5472.CAN-18-0030 29967261

[217] Tuveson D Clevers H. Cancer Modeling Meets Human Organoid Technology. Science (2019) 364(6444):952–5. doi: 10.1126/science.aaw6985 31171691

[218] Grunblatt E Wu N Zhang H Liu X Norton JP Ohol Y. MYCN Drives Chemoresistance in Small Cell Lung Cancer While USP7 Inhibition can Restore Chemosensitivity. Genes Dev (2020) 34(17-18):1210–26. doi: 10.1101/gad.340133.120 PMC7462062 32820040

[219] Li L Ng SR Colón CI Drapkin BJ Hsu PP Li Z. Identification of DHODH as a Therapeutic Target in Small Cell Lung Cancer. Sci Transl Med (2019) 11(517). doi: 10.1126/scitranslmed.aaw7852 PMC7401885 31694929

[220] Yamauchi T. Exploration of Novel Therapeutic Targets in Acute Myeloid Leukemia via Genome-Wide CRISPR Screening. Rinsho Ketsueki (2019) 60(7):810–7. doi: 10.11406/rinketsu.60.810 31391371

[221] Lin KH Rutter JC Xie A Pardieu B Winn ET Bello RD. Using Antagonistic Pleiotropy to Design a Chemotherapy-Induced Evolutionary Trap to Target Drug Resistance in Cancer. Nat Genet (2020) 52(4):408–17. doi: 10.1038/s41588-020-0590-9 PMC7398704 32203462

[222] Bharathy N Berlow NE Wang E Abraham J Settelmeyer TP Hooper JE. Preclinical Rationale for Entinostat in Embryonal Rhabdomyosarcoma. Skelet Muscle (2019) 9(1):12. doi: 10.1186/s13395-019-0198-x 31113472 PMC6528217

[223] Szlachta K Kuscu C Tufan T Adair SJ Shang S Michaels AD. CRISPR Knockout Screening Identifies Combinatorial Drug Targets in Pancreatic Cancer and Models Cellular Drug Response. Nat Commun (2018) 9(1):4275. doi: 10.1038/s41467-018-06676-2 30323222 PMC6189038

[224] Michels BE Mosa MH Streibl BI Zhan T Menche C Abou-El-Ardat K. Pooled In Vitro and In Vivo CRISPR-Cas9 Screening Identifies Tumor Suppressors in Human Colon Organoids. Cell Stem Cell (2020) 26(5):782–92.e7. doi: 10.1016/j.stem.2020.04.003 32348727

[225] Mis M O'Brien S Steinhart Z Lin S Hart T Moffat J. IPO11 Mediates βcatenin Nuclear Import in a Subset of Colorectal Cancers. J Cell Biol (2020) 219(2):e201903017. doi: 10.1083/jcb.201903017 31881079 PMC7041691

[226] Green S Dam MS Svendsen MN. Mouse Avatars of Human Cancers: The Temporality of Translation in Precision Oncology. Hist Philos Life Sci (2021) 43(1):27. 33620596 10.1007/s40656-021-00383-w

[227] Letrado P de Miguel I Lamberto I Díez-Martínez R Oyarzabal J. Zebrafish: Speeding Up the Cancer Drug Discovery Process. Cancer Res (2018) 78(21):6048–58. doi: 10.1158/0008-5472.CAN-18-1029 30327381

[228] Wu Q Kumar N Velagala V Zartman JJ. Tools to Reverse-Engineer Multicellular Systems: Case Studies Using the Fruit Fly. J Biol Eng (2019) 13:33. doi: 10.1186/s13036-019-0161-8 31049075 PMC6480878

[229] Port F Strein C Stricker M Rauscher B Heigwer F Zhou J. A Large-Scale Resource for Tissue-Specific CRISPR Mutagenesis in Drosophila. Elife (2020) 9:e53865. doi: 10.7554/eLife.53865 32053108 PMC7062466

[230] Keatinge M Tsarouchas TM Munir T Porter NJ Larraz J Gianni D. CRISPR gRNA Phenotypic Screening in Zebrafish Reveals Pro-Regenerative Genes in Spinal Cord Injury. PloS Genet (2021) 17(4):e1009515. doi: 10.1371/journal.pgen.1009515 33914736 PMC8084196

